# A Theoretical Study of Absorption–Emission Asymmetry from Franck–Condon–Herzberg–Teller Interference and Duschinsky Mixing

**DOI:** 10.1021/acs.jpca.6c04260

**Published:** 2026-10-13

**Authors:** Cedric Poon, Chun Hey Siu

**Affiliations:** † Department of Chemistry, 4615 Imperial College London, London SW7 2AZ, U.K.; ‡ Department of Chemistry, 58207 The Hong Kong University of Science and Technology, Clear Water Bay, Hong Kong 999077, China

## Abstract

Herzberg–Teller (HT) coupling beyond the Franck–Condon (FC) approximation can break the mirror-image relation between absorption and emission vibronic spectra through interference between the FC and HT transition amplitudes. Here, we analyze the geometry dependence of this FC–HT interference using a displaced harmonic-oscillator model and extend the treatment perturbatively to Duschinsky rotation. In the separable equal-frequency limit, the FC and HT vibrational integrals are strictly proportional, reducing the asymmetry to a single variable κ that separates into an electronic ratio of HT and shifted FC prefactors and a nuclear ratio of vibrational spacing to signed displacement. For HT-active totally symmetric modes, reversing the displacement reverses the interference direction, identifying signed displacement, rather than the Huang–Rhys factor, as the governing nuclear variable. For nondegenerate, nontotally symmetric modes, interference emerges under symmetry-breaking displacement, with its direction fixed by the vibrational quantum change alone. The normalized asymmetry depends only on vibrational spacing, allowing an observed progression to probe the same universal dependence at fixed geometry. Applied to experimental phenanthrene spectra, this progression-based analysis recovers strikingly similar first-order HT information from the absorption–emission asymmetry. Finally, the leading Duschinsky correction decomposes into geometric and electronic pathways built from the same one-mode quantities as the separable limit, showing that mode mixing redistributes FC and HT character between coordinates rather than introducing a new electronic quantity.

## Graphical abstract

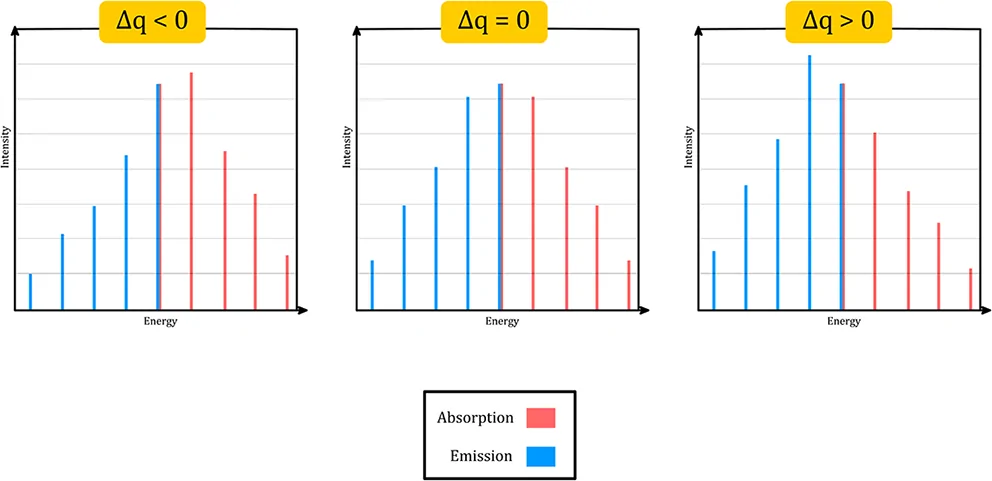

## 1 Introduction

The Franck–Condon (FC) approximation provides the standard starting point for interpreting vibrational structure in electronic spectra.^1^ In its simplest form, transition intensities are determined by vibrational overlap integrals evaluated with a coordinate-independent electronic transition dipole moment. This description is often successful for strongly allowed transitions, but it becomes inadequate for weak bands and nominally forbidden transitions, where the transition dipole varies appreciably with nuclear geometry. These cases are naturally treated by the incorporation of Herzberg–Teller (HT) coupling,^2,3^ which accounts for the first-order dependence of the transition dipole moment on nuclear displacement and thereby enables intensity borrowing into symmetry-forbidden channels.

A widely recognized consequence is that, once HT terms are included, the mirror symmetry between absorption and emission spectra, expected in the FC picture, need not survive.^4–15^ The resulting asymmetry is more than a perturbative change in peak intensity, as it can alter apparent vibronic progressions, redistribute intensity across a band, and complicate the structural interpretation of absorption and fluorescence spectra.

Experimental departures from absorption–emission mirror symmetry were identified early on in aromatic systems, most notably in phenanthrene, and were attributed to interference between the FC and HT transition amplitudes.^16–18^ Subsequent studies of polycyclic aromatic hydrocarbons reinforced this interpretation and emphasized the particular importance of totally symmetric promoting modes.^7,11^ More recently, Kundu, Roy, and coworkers placed the parity-controlled origin of this effect on a general footing within the displaced harmonic-oscillator limit.^8,19^ Their analysis established that the direction of FC–HT interference is governed jointly by the relative signs of the FC and HT amplitudes and by the change in vibrational quanta.

These results provide an essential limiting-case description of mirror-symmetry breaking, but they are not yet sufficient for assigning spectral asymmetry in molecular systems. First, a Huang–Rhys factor characterizes the magnitude of a displacement but not its direction. For totally symmetric coordinates, distortions of opposite sign correspond to physically inequivalent molecular structures and therefore need not produce equivalent FC–HT interference patterns.^20,21^ Second, nontotally symmetric HT-active modes have received comparatively little attention because FC–HT interference is commonly assumed to be absent when the FC contribution vanishes at a high-symmetry reference geometry. A symmetry-breaking distortion can, however, activate an FC contribution in a dipole component that is forbidden at the parent geometry, thereby reopening interference channels that do not exist in the high-symmetry limit.^22,23^ Most importantly, previous analytical treatments generally suppress Duschinsky rotation in order to isolate the idealized interference mechanism. This simplification is not neutral because Duschinsky mixing couples nominally distinct vibrational coordinates, alters the composition of FC and HT amplitudes, and can modify the relation between absorption and emission transition strengths. Moreover, neglecting such mixing is not strictly consistent with a complete treatment of HT coupling, since vibronic interactions can themselves induce rotations in normal-coordinate space.^3,24^

In this work, we present a theoretical and computational study of FC–HT absorption–emission asymmetry. We first establish what controls the asymmetry within the adiabatic-shift (AS) displaced-oscillator framework, examining its dependence on the signed displacement along a normal coordinate, on the electronic prefactors, and on the vibronic levels involved in the transition. This account is then generalized to combination transitions, where two coordinates are excited simultaneously and the asymmetry acquires qualitative features with no single-mode counterpart. With the AS behavior established as a baseline, the same description is finally carried beyond the separable limit into Duschinsky mixing, which is treated not as a separate problem but as a perturbation about the unrotated limit, with the mixing angle as the expansion parameter. This allows the question of whether mode mixing constitutes an independent route to mirror-symmetry breaking, or instead acts through the interference structure already present, to be posed directly and the two-mode case to be connected to the many-mode situation encountered in molecular spectra. It should be noted that the harmonic treatment isolates the desired mechanism, but contributions from other effects, like anharmonicity, can often equal or exceed FC–HT interference. These other contributions are not ranked here.

## 2 Theoretical Framework

### 2.1 Background

Consider a transition from the u-th vibrational state of the ground electronic state (GS) to the w-th vibrational state of an excited electronic state (ES). The corresponding transition dipole moment matrix element is

1 $$M_{\mathrm{FC}}\left\langle \chi_{\mathrm{GS:u}}|\chi_{\mathrm{ES:w}}\right\rangle +\overset{3N-6}{\underset{n=1}{\sum }}M_{\mathrm{HT}}^{\left(n\right)}\left\langle \chi_{\mathrm{GS:u}}\left|q_{n}-q_{\mathrm{eq},n}\right|\chi_{\mathrm{ES:w}}\right\rangle$$

where χ denotes a harmonic-oscillator wavefunction, *q*_*n*_ is the *n*-th normal coordinate, M_FC_ is the coordinate-independent electronic transition dipole moment, and $M_{\mathrm{HT}}^{\left(n\right)}$ is the first derivative of the electronic transition dipole moment with respect to *q*_*n*_.

For clarity, the discussion here is initially restricted to a single normal mode, whose coordinate is written as *q* with the mode index dropped. Within the displaced harmonic-oscillator framework, the ground- and excited-state potential-energy curves differ only in the positions of their minima, *q*_eq,GS_ and *q*_eq,ES_. It is therefore convenient to introduce the midpoint-shifted coordinate

2 $$q_{M}=q-\frac{q_{\mathrm{eq},\mathrm{GS}}+q_{\mathrm{eq},\mathrm{ES}}}{2}$$

and the displacement between the two minima,

3 $$\Delta q=q_{\mathrm{eq},\mathrm{ES}}-q_{\mathrm{eq},\mathrm{GS}}$$

The transition dipole moment can thus be expressed symmetrically about the midpoint. This introduces the shifted transition-dipole term M̃_FC_,

4 $$M\left(q\right)=M_{\mathrm{FC}}+M_{\mathrm{HT}}\left(q-q_{\mathrm{eq},\mathrm{ES}}\right)=\left(M_{\mathrm{FC}}-\frac{\Delta q}{2}M_{\mathrm{HT}}\right)+M_{\mathrm{HT}}q_{M}={\mathrm{M̃}}_{\mathrm{FC}}+M_{\mathrm{HT}}q_{M}$$

To discuss mirror symmetry, we compare cross-swap pairs (CSPs), defined as pairs of transitions in which the electronic labels are exchanged while the vibrational quantum numbers are retained:

$$\begin{array}{ll} \mathrm{absorption:} & |\Psi_{\mathrm{GS:u}}\rangle \to \left|\Psi_{\mathrm{ES:w}}\right\rangle \\ \mathrm{emission:} & \left|\Psi_{\mathrm{ES:u}}\right\rangle \to \left|\Psi_{\mathrm{GS:w}}\right\rangle \end{array}$$

The transition dipole moments for the two members of the CSP are

5 $$A={\mathrm{M̃}}_{\mathrm{FC}}\left\langle \chi_{\mathrm{GS:u}}|\chi_{\mathrm{ES:w}}\right\rangle +M_{\mathrm{HT}}\left\langle \chi_{\mathrm{GS:u}}\left|q_{M}\right|\chi_{\mathrm{ES:w}}\right\rangle$$

6 $$E={\mathrm{M̃}}_{\mathrm{FC}}\left\langle \chi_{\mathrm{ES:u}}|\chi_{\mathrm{GS:w}}\right\rangle +M_{\mathrm{HT}}\left\langle \chi_{\mathrm{ES:u}}\left|q_{M}\right|\chi_{\mathrm{GS:w}}\right\rangle$$

Using the midpoint coordinate, the FC and HT vibrational integrals for absorption can be written in the symmetric forms

7 $$F^{\left(\mathrm{u;w}\right)}\equiv \left\langle \chi_{\mathrm{GS:u}}|\chi_{\mathrm{ES:w}}\right\rangle =\int_{-\infty }^{\infty }\chi_{u}\left(q_{M}+\frac{\Delta q}{2}\right)\chi_{w}\left(q_{M}-\frac{\Delta q}{2}\right)\;dq_{M}$$

8 $$H^{\left(\mathrm{u;w}\right)}\equiv \left\langle \chi_{\mathrm{GS:u}}\left|q_{M}\right|\chi_{\mathrm{ES:w}}\right\rangle =\int_{-\infty }^{\infty }\chi_{u}\left(q_{M}+\frac{\Delta q}{2}\right)q_{M}\chi_{w}\left(q_{M}-\frac{\Delta q}{2}\right)\;dq_{M}$$

Because harmonic-oscillator wavefunctions have definite parity, the FC and HT integrals transform oppositely under *q*_M_ → −*q*_M_. For a given Δν = w – u, defining σ = (−1)^Δν^, the members of a CSP satisfy

9 $$F^{\left(\mathrm{w;u}\right)}=\sigma F^{\left(\mathrm{u;w}\right)}H^{\left(\mathrm{w;u}\right)}=-\sigma H^{\left(\mathrm{u;w}\right)}$$

From this relation, it is clear that the relative sign of the FC and HT contributions must be opposite, regardless of the value of Δν. Constructive FC–HT interference in one member of a CSP therefore corresponds to destructive interference in the other.

To quantify the direction and magnitude of this interference, the asymmetry factor is defined as

10 $$\zeta \equiv \frac{\left|A{|}^{2}-\right|E{|}^{2}}{\left|A{|}^{2}+\right|E{|}^{2}}=\frac{2{\mathrm{M̃}}_{\mathrm{FC}}M_{\mathrm{HT}}F^{\left(\mathrm{u;w}\right)}H^{\left(\mathrm{u;w}\right)}}{{\left({\mathrm{M̃}}_{\mathrm{FC}}F^{\left(\mathrm{u;w}\right)}\right)}^{2}+{\left(M_{\mathrm{HT}}H^{\left(\mathrm{u;w}\right)}\right)}^{2}}$$

Equation 10 shows that absorption–emission asymmetry arises only when both FC and HT pathways are nonzero and interfere. Its sign is determined jointly by the electronic prefactors and the relative signs of the FC and HT vibrational integrals.

### 2.2 Reduction of the Asymmetry Factor

Equations 9–10 restate, in the midpoint convention, the parity structure established by Kundu and Roy within the displaced harmonic-oscillator limit. In that form, the asymmetry factor still depends on the two vibrational integrals separately, and hence, apparently, on the individual vibronic levels u and w. We now show that this dependence is illusory.

In the displaced-oscillator limit, the FC and HT integrals are not only related by parity but also strictly proportional, with a proportionality constant fixed by the vibrational spacing and the displacement alone. The asymmetry factor consequently collapses onto a universal single-variable curve, from which the sign rules used throughout Section 4 follow as corollaries.

For equal ground- and excited-state frequencies, the FC overlap integral of eq 7 admits the closed form

11 $$F^{\left(\mathrm{u;w}\right)}=\exp\left(-\frac{{\Delta q}^{2}}{4}\right)\sqrt{\frac{u!}{w!}}{\left(-\frac{\Delta q}{\sqrt{2}}\right)}^{\Delta \nu }L_{u}^{\left(\Delta \nu \right)}\left(\frac{{\Delta q}^{2}}{2}\right)$$

where $L_{u}^{\left(\Delta \nu \right)}$ is an associated Laguerre polynomial and Δν = w – u ≥ 0; the case Δν < 0 is recovered from eq 9 and is treated below. The corresponding HT integral of eq 8 is obtained by expressing *q*_M_ in terms of the harmonic-oscillator ladder operators, giving

12 $$H^{\left(\mathrm{u;w}\right)}=\frac{1}{\sqrt{2}}\left[\sqrt{u}F^{\left(u-1;w\right)}+\sqrt{u+1}F^{\left(u+1;w\right)}\right]-\frac{\Delta q}{2}F^{\left(\mathrm{u;w}\right)}$$

The HT integral is therefore not independent of the FC integrals but is built from the two overlaps neighboring F^(u;w)^ in the initial vibrational level.

Setting w = u + Δν and writing the neighboring FC overlaps in eq 12 as Manneback ratios relative to F^(u;w)^ gives

13 $$H^{\left(\mathrm{u;w}\right)}=\left(-\frac{\Delta q}{2}\frac{L_{u-1}^{\left(\Delta \nu +1\right)}\left(\alpha \right)}{L_{u}^{\left(\Delta \nu \right)}\left(\alpha \right)}-\frac{u+1}{\Delta q}\frac{L_{u+1}^{\left(\Delta \nu -1\right)}\left(\alpha \right)}{L_{u}^{\left(\Delta \nu \right)}\left(\alpha \right)}-\frac{\Delta q}{2}\right)F^{\left(\mathrm{u;w}\right)}$$

where α = Δ*q* ^2^/2 and $L_{-1}^{\left(\Delta \nu +1\right)}\equiv 0$ . Both ratios retain an explicit dependence on the absolute level u. This dependence cancels upon applying the associated Laguerre recurrence relation

14 $$\alpha L_{u-1}^{\left(\Delta \nu +1\right)}\left(\alpha \right)+\left(u+1\right)L_{u+1}^{\left(\Delta \nu -1\right)}\left(\alpha \right)=\left(\Delta \nu -\alpha \right)L_{u}^{\left(\Delta \nu \right)}\left(\alpha \right)$$

which upon division by $\Delta qL_{u}^{\left(\Delta \nu \right)}\left(\alpha \right)$ and use of $\frac{\alpha }{\Delta q}=\frac{\Delta q}{2}$ implies that

15 $$\frac{\Delta q}{2}\frac{L_{u-1}^{\left(\Delta \nu +1\right)}\left(\alpha \right)}{L_{u}^{\left(\Delta \nu \right)}\left(\alpha \right)}+\frac{u+1}{\Delta q}\frac{L_{u+1}^{\left(\Delta \nu -1\right)}\left(\alpha \right)}{L_{u}^{\left(\Delta \nu \right)}\left(\alpha \right)}=\frac{\Delta \nu }{\Delta q}-\frac{\Delta q}{2}$$

Upon substitution of eq 15 into eq 13, the two terms in $\frac{\Delta q}{2}$ cancel and leave the compact proportionality

16 $$H^{\left(\mathrm{u;w}\right)}=\gamma F^{\left(\mathrm{u;w}\right)},,\gamma =-\frac{\Delta \nu }{\Delta q}$$

Equation 16 was derived for Δν ≥ 0 but extends to negative spacings without further work. Applying the CSP parity relations of eq 9 to the ratio gives

17 $$\frac{H^{\left(\mathrm{w;u}\right)}}{F^{\left(\mathrm{w;u}\right)}}=\frac{-\sigma H^{\left(\mathrm{u;w}\right)}}{\sigma F^{\left(\mathrm{u;w}\right)}}=-\gamma =-\frac{u-w}{\Delta q}$$

which is eq 16 with Δν replaced by u – w. The identity therefore holds for either sign of Δν, and the sign reversal of γ between the two members of a CSP is precisely the opposite-parity relation of eq 9 expressed as a single scalar.

The consequences for the asymmetry factor are immediate. Substituting eq 16 into eq 10 cancels the common factor (F^(u;w)^)^2^ between the numerator and denominator,

18 $$\zeta =\frac{2{\mathrm{M̃}}_{\mathrm{FC}}M_{\mathrm{HT}}\gamma }{{{\mathrm{M̃}}_{\mathrm{FC}}}^{2}+{M_{\mathrm{HT}}}^{2}\gamma^{2}}$$

so that all vibrational information enters through γ alone. Introducing the reduced interference variable

19 $$\kappa \equiv \frac{M_{\mathrm{HT}}\gamma }{{\mathrm{M̃}}_{\mathrm{FC}}}=-\frac{M_{\mathrm{HT}}}{{\mathrm{M̃}}_{\mathrm{FC}}}\frac{\Delta \nu }{\Delta q}$$

eq 18 reduces to the universal form

20 $$\zeta =\frac{2\kappa }{1+\kappa^{2}}$$

In the displaced-oscillator limit at equal ground- and excited-state frequencies, the entire asymmetry problem therefore collapses into a single one-parameter curve. The four quantities that enter κ separate cleanly into an electronic ratio, $\frac{M_{\mathrm{HT}}}{{\mathrm{M̃}}_{\mathrm{FC}}}$ , and a nuclear one, $\frac{\Delta \nu }{\Delta q}$ .

It should be noted that eq 11 and hence eq 16 assume equal ground- and excited-state frequencies; a frequency change introduces an additional squeezing contribution to the overlap, and γ acquires a dependence on u that no longer cancels. The reduction of the asymmetry to the single variable κ is also specific to the separable displaced-oscillator limit. Even when Duschinsky mixing is introduced in Section 4.4, γ_*X*_ remains well-defined for each coordinate, but is no longer sufficient by itself to describe the asymmetry. The leading correction additionally involves the displacement response of the FC overlap, and combinations of these one-mode quantities across the mixed coordinates. Within the separable equal-frequency limit, however, eq 20 governs the behavior of the asymmetry completely, and the results of Section 4 follow from it as corollaries.

## 3 Computational Details

To keep the notation unambiguous, each normal mode is labeled α­(*n*)^ν^, where *n* is the irreducible representation, α ranks modes of the same symmetry by increasing frequency, and ν is the vibrational quantum number. Since mirror-related members of cross-swap pairs (CSPs) are compared throughout, these pairs are denoted as α­(*n*)^u→w^, representing the absorption transition S_0_[u] → S_1_[w] and the corresponding emission transition S_1_[u] → S_0_[w] in mode α­(*n*).

Density functional theory (DFT) and its time-dependent extension (TD-DFT) were used to carry out the electronic structure calculations for the ground and excited electronic states, respectively. All calculations were performed with Gaussian 16.^25^ The ωB97X-D functional^26^ was used in conjunction with the aug-cc-pVDZ basis set.^27^ The time-independent FC–HT implementation developed by Barone and co-workers^28,29^ was used for full-dimensional reference calculations and for extracting the vibronic quantities required to validate the reduced models.

The calculations were organized into two related protocols. The first protocol uses the adiabatic-shift (AS) displaced-oscillator limit, in which a single normal-mode basis is retained and Duschinsky rotation is completely suppressed. This protocol isolates the geometry dependence of FC–HT interference along individual normal coordinates. The second protocol uses a two-mode Duschinsky model, in which the active normal coordinates of the two electronic states are related by an explicit rotation matrix. The two protocols share the same electronic-structure input and differ only in the vibrational coordinate transformation used to evaluate the FC and HT overlap integrals.

### 3.1 Electronic-Structure Parametrization

TD-DFT calculations were performed by requesting the lowest six singlet excited states, with the first excited singlet state, S_1_, selected as the target state throughout. The FC electronic prefactor, M_FC_, was obtained from the length-gauge electric transition dipole moment of the selected transition.

The HT electronic prefactors are the first derivatives of the electronic transition dipole moment with respect to the dimensionless normal coordinates, so that M_FC_, M̃_FC_, and M_HT_ share units of *e·a*_0_. The Cartesian derivatives were collected in a matrix *P̂* of dimension 3*N* × 3, whose rows follow the nuclear Cartesian-coordinate ordering and whose columns correspond to the *x*, *y,* and *z* components of the transition dipole moment. Projection onto the S_0_ normal-mode basis gives

21 $${\mathrm{M̂}}_{\mathrm{HT}}\;={\mathrm{P̂}}^{T}\mathrm{N̂}$$

where $\mathrm{N̂}\equiv {\mathrm{M̂}}^{-\frac{1}{2}}\mathrm{L̂}{\mathrm{\Omega ̂}}^{-\frac{1}{2}}$ is the matrix that carries dimensionless normal coordinates into Cartesian displacements, with L̂ the orthonormal mass-weighted normal-mode matrix, M̂ the atomic masses in atomic units, and Ω̂ the harmonic frequencies in hartrees. Its k-th column $N_{k}$ is the Cartesian displacement produced by a unit change in *q*_k_.

The molecular orientations used to define the Cartesian transition dipole components, together with additional displacement scans for modes and systems not shown in the main text, are provided in the Supporting Information.

### 3.2 Adiabatic-Shift Displaced-Oscillator Model

In the AS treatment, as illustrated by Figure 1, the excited-state potential-energy surface is represented in the ground-state normal-mode basis. The Duschinsky matrix is therefore set to the identity, so that the two harmonic potentials share a common set of normal coordinates and differ only by a displacement along the selected mode. This construction suppresses mode mixing and provides the separable reference limit in which absorption–emission asymmetry can be assigned directly to FC–HT interference.

**Figure 1:**
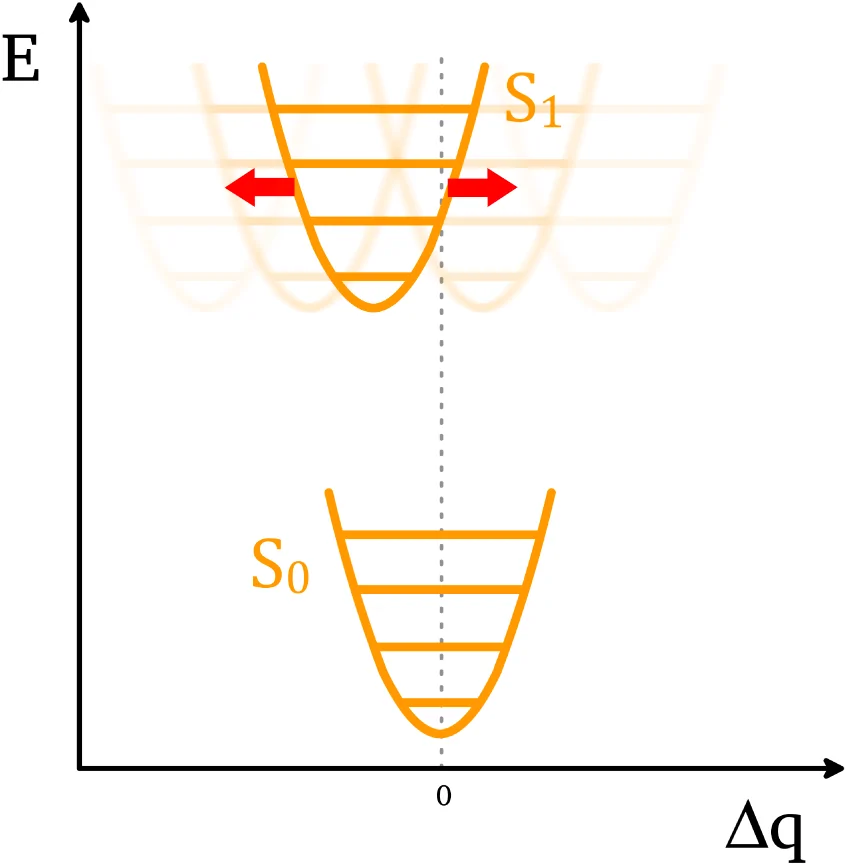
Schematic of the fixed-reference adiabatic-shift displaced harmonic-oscillator model used for the displacement scans. Ground- and excited-state harmonic potentials of equal curvature are represented in a common normal-coordinate basis, with the excited-state minimum displaced by a signed amount Δ*q* along the selected coordinate. The displacement is varied in both directions while the reference normal modes, harmonic frequencies, and first-order electronic transition-moment parameters are held fixed. The resulting scans therefore represent a single linear Herzberg–Teller expansion about the reference geometry, with only the relative displacement of the harmonic potentials varied.

The ground-state geometry was first optimized to obtain the reference geometry **Q**_0_. The transformation matrix N̂ and harmonic frequencies were obtained from a subsequent frequency calculation. For a selected mode k, the signed dimensionless displacement Δ*q*_k_ defines the position of the displaced excited-state harmonic minimum relative to the reference geometry,

22 $$Q_{\mathrm{ES}}\left({\Delta q}_{k}\right)=Q_{0}+{\Delta q}_{k}N_{k}$$

In the main analysis, this relation parametrizes the displacement of the harmonic model, and the electronic transition-moment parameters are not recalculated at the displaced geometries.

Instead, the displacement scans employ a single first-order expansion of the electronic transition dipole about the reference geometry **Q**_0_. The reference FC prefactor, $M_{\mathrm{FC}}^{\left(0\right)}$ , and the corresponding HT derivative, $M_{\mathrm{HT},k}^{\left(0\right)}$ , are evaluated once at **Q**_0_ and retained throughout the scan. Along mode *k*, the transition dipole is therefore represented globally as

23 $$M\left(q_{k}\right)=M_{\mathrm{FC}}^{\left(0\right)}+M_{\mathrm{HT},k}^{\left(0\right)}q_{k}$$

with $M_{\mathrm{HT},k}^{\left(0\right)}$ independent of Δ*q*_k_. Expressing the same expansion in the midpoint convention of eq 4 gives

24 $${\mathrm{M̃}}_{\mathrm{FC}}\left({\Delta q}_{k}\right)=M_{\mathrm{FC}}^{\left(0\right)}+\frac{{\Delta q}_{k}}{2}M_{\mathrm{HT},k}^{\left(0\right)}$$

so that the shifted FC prefactor varies linearly with displacement while the HT prefactor remains constant.

The reference normal-mode basis, harmonic frequencies, and vibrational wavefunctions are held fixed throughout the scan, such that the main text results correspond to a single fixed-reference, globally linear HT model. Second- and higher-order derivatives of the electronic transition dipole moment, frequency changes, Duschinsky rotation, and anharmonicity are not included in the AS scans.

For comparison, the Supporting Information presents both the fixed-reference treatment and the geometry-reparametrized procedure, in which the transition dipole and its first derivative are reevaluated at finite displacements while the reference harmonic basis is retained. This comparison quantifies the departure from the reference linear form.

The displacement analysis was restricted to modes for which a rectilinear normal-coordinate description remains physically meaningful over the range considered. Modes with significant torsional character were therefore excluded. Nevertheless, the AS scans remain idealized harmonic constructions and do not include finite-amplitude mode coupling or anharmonicity.

### 3.3 Two-Mode Duschinsky-Rotation Model

The two-mode Duschinsky model extends the AS framework by introducing an explicit rotation between the ground- and excited-state normal-mode bases, allowing mode mixing and its effect on FC–HT interference to be studied in a controlled setting. The Duschinsky matrix is parametrized as a 2 × 2 rotation matrix with a manually varied angle θ.

25 $$Ĵ=\left[\begin{array}{cc} \cos\theta & \sin\theta \\ -\sin\theta & \cos\theta \end{array}\right]$$

Independent geometry optimizations and harmonic frequency calculations were performed for the ground and excited electronic states. The electronic prefactors, shift vector **K**, and reduced frequencies were then extracted and used to compute the FC and HT overlap integrals.

At each value of θ, the Sharp–Rosenstock matrices *Â*, **B**, *Ĉ*, **D**, and *Ê* and the zero-phonon overlap integral $I_{\mathrm{FC}}^{\left(0\to 0\right)}$ were computed from *Ĵ*(θ), the shift vector, and the reduced frequencies ω_*k*_ following the same internal formulas used by Gaussian 16:

26 $$\mathrm{X̂}={\left({Ĵ}^{T}{Ĝ}^{\prime }Ĵ+{Ĝ}^{\prime \prime }\right)}^{-1},,Ŷ=Ĵ\mathrm{X̂}{Ĵ}^{T}$$

27 $$Â=2{Ĝ}^{\prime \frac{1}{2}}Ŷ{Ĝ}^{\prime \frac{1}{2}}-Î,,B=-2{Ĝ}^{\prime \frac{1}{2}}\left(Ŷ{Ĝ}^{\prime }-Î\right)K$$

28 $$Ĉ=2{Ĝ}^{\prime \prime \frac{1}{2}}\mathrm{X̂}{Ĝ}^{\prime \prime \frac{1}{2}}-Î,,D=-2{Ĝ}^{\prime \prime \frac{1}{2}}\mathrm{X̂}{Ĵ}^{T}{Ĝ}^{\prime }K$$

29 $$Ê=4{Ĝ}^{\prime \prime \frac{1}{2}}\mathrm{X̂}{Ĵ}^{T}{Ĝ}^{\prime \frac{1}{2}}$$

where ${Ĝ}^{\prime }=\mathrm{diag}\left(\omega_{k}^{S_{0}}\right)$ and ${Ĝ}^{\prime \prime }=\mathrm{diag}\left(\omega_{k}^{S_{1}}\right)$ are diagonal matrices of the initial- and final-state reduced frequencies, respectively.

30 $$I_{\mathrm{FC}}^{\left(0\to 0\right)}=2^{N/2}{[\det(G^{\prime }G^{\prime \prime })]}^{\frac{1}{4}}{[\frac{\det(Ĵ)}{\det({\mathrm{X̂}}^{-1})}]}^{\frac{1}{2}},\exp\left[-\frac{1}{2}K^{T}{Ĝ}^{\prime }K+\frac{1}{2}K^{T}{Ĝ}^{\prime }Ŷ{Ĝ}^{\prime }K\right]$$

The FC integrals were then computed as

31 $$F^{\left(u;w\right)}=\frac{1}{\sqrt{w_{k}}}[\frac{d_{k}}{\sqrt{2}}F^{\left(u;w-e_{k}\right)}+\sqrt{w_{k}-1}C_{kk}F^{\left(u;w-2e_{k}\right)}+\underset{j<k}{\sum }\sqrt{w_{j}}C_{kj}F^{\left(u;w-e_{k}-e_{j}\right)}+\frac{1}{2}\underset{i}{\sum }\sqrt{u_{i}}E_{ki}F^{\left(u-e_{i};w-e_{k}\right)}]$$

with the HT integrals calculated using the ladder operator expression.

## 4 Results and Discussion

### 4.1 Spectral Signature of FC–HT Interference

Before analyzing how FC–HT interference evolves with geometry and Duschinsky rotation, it is useful to identify its direct spectral signature. Figure 2 shows simulated absorption and emission spectra of pyrene computed at the FC and FC–HT levels. In the pure FC regime, absorption and emission are mirror-related because the transition strength is determined by |M_FC_F^(u;w)^|^2^. Thus, even if the FC overlap changes sign between the two members of a CSP, as described by σ in eq 9, this phase is removed when the square modulus is taken, and no absorption–emission asymmetry is produced. Equivalently, M_HT_ = 0 gives κ = 0 in eq 19 and hence ζ = 0 identically.

**Figure 2:**
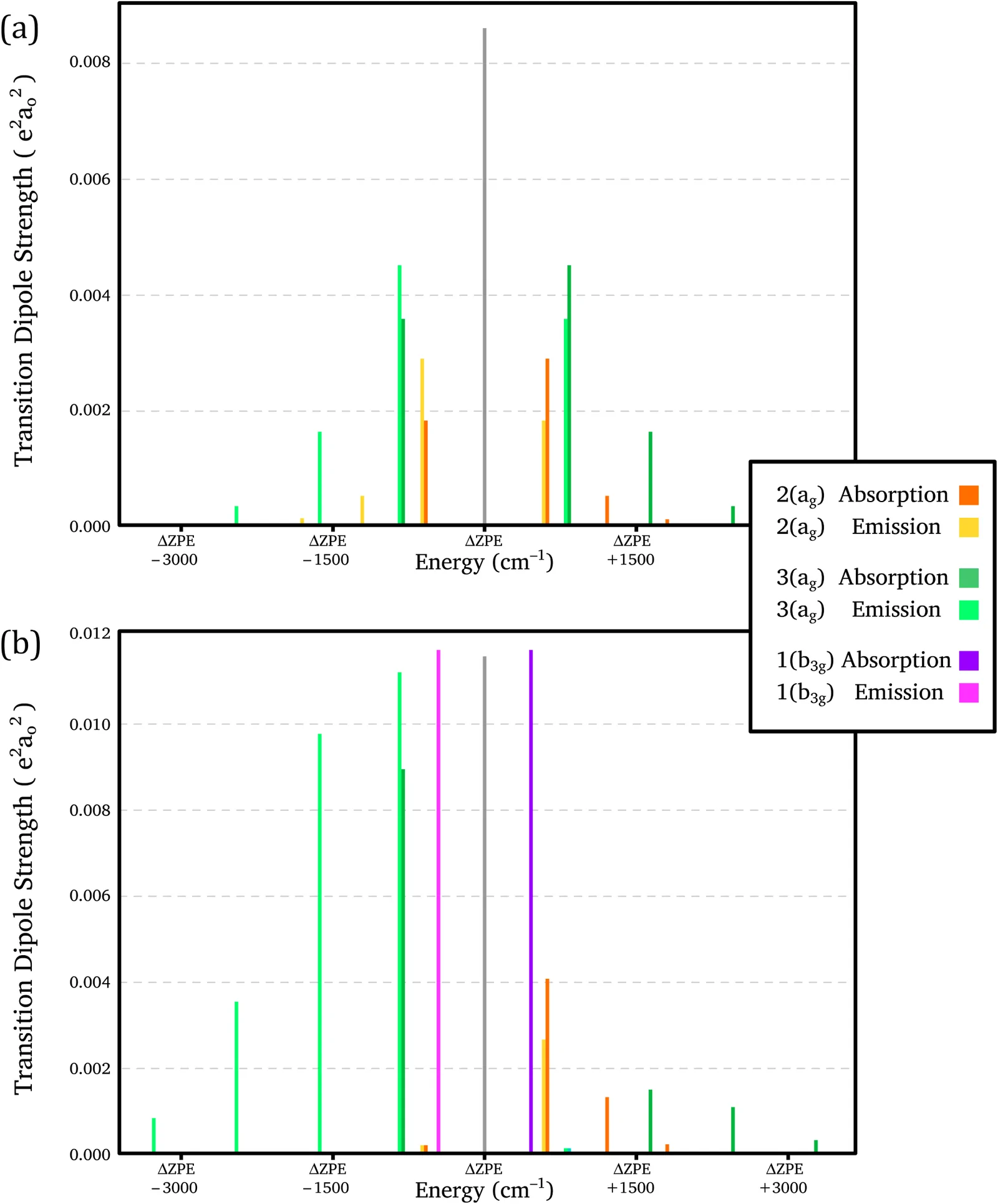
Simplified simulated pyrene spectra computed at the (a) FC and (b) FC–HT levels. Only single-mode transitions are included for modes 2­(a_g_) (Δ*q* ≈ −0.63), 3­(a_g_) (Δ*q* ≈ 0.91), and 1­(b_3g_) (Δ*q* = 0).

This changes when the transition moment is expanded to first order in the nuclear coordinate. The resulting FC–HT spectrum contains an interference contribution arising from the simultaneous presence of FC and HT transition pathways. Unlike the pure FC or pure HT terms, this interference contribution is signed, and can therefore enhance or suppress a vibronic transition depending on the relative phase of the two amplitudes. Consequently, when the FC–HT cross term amplifies the intensity of one member of a CSP, it suppresses the mirror-related member. This redistribution is visible for the displaced 2­(a_g_) and 3­(a_g_) progressions in Figure 2b. By contrast, the 1­(b_3g_) mode remains symmetric because its normal coordinate is undisplaced. In this limit, the ground- and excited-state vibrational bases coincide perfectly and FC intensity is restricted to the zero-phonon line by orthogonality, while vibronic sidebands are purely HT in origin. Since the FC and HT pathways do not contribute simultaneously to the same sideband, no FC–HT interference can occur.

The displaced progressions also show that the direction of the asymmetry reverses across the zero-phonon line. For a fixed mode, vibronic lines on one side of the electronic-origin transition are amplified in absorption and damped in emission, whereas lines on the opposite side display the opposite behavior. The two sides correspond to opposite signs of Δν, and the reversal follows directly from eq 19, where κ is linear in Δν, and ζ has the sign of κ. Collecting the remaining factors entering κ, the direction of the interference at any fixed geometry can be written as

32 $$\mathrm{sgn}\left(\zeta \right)=-\mathrm{sgn}\left(\Delta \nu \right)\;\mathrm{sgn}\left(\Delta q\right)\;\mathrm{sgn}\left({\mathrm{M̃}}_{\mathrm{FC}}M_{\mathrm{HT}}\right)$$

### 4.2 Single-Mode Transitions

We begin by considering transitions in which only a single HT-active normal mode changes its vibrational quanta, deferring combination bands to Section 4.3. Within this restricted setting, the geometry dependence of FC–HT interference can be traced directly to the parity structure of the vibronic overlap integrals and the displacement dependence of the midpoint-shifted FC contribution.

#### 4.2.1 Totally Symmetric HT-Active Modes

For a totally symmetric coordinate, the distortion preserves the full parent point group, and in the resulting one-dimensional deformation space there is no nontrivial symmetry operation that identifies +Δ*q* with −Δ*q*.^20,21^ Physically, this means that displacements toward +Δ*q* and −Δ*q* must always produce different molecular structures, so the two directions must be structurally inequivalent and need not carry the same interference character.

It is worth stressing that, while the sign convention for Δ*q* is completely arbitrary, the underlying physics is not. If the coordinate is redefined by Δ*q* → −Δ*q*, then M_HT_, as a derivative taken along that coordinate, must also change sign. Since these terms appear only as either Δ*q*M_HT_ or M_HT_^2^ in eq 10, the sign of the asymmetry factor is invariant with respect to the assignment of Δ*q*. The fact that these combinations arise naturally from the derivation, rather than being imposed *ad hoc*, speaks to the fundamental character of the interference.

The left panels of Figure 3 show the absorption $|A{|}^{2}$ and emission $|E{|}^{2}$ transition dipole strengths as functions of Δ*q*. In all cases, both curves intersect exactly at Δ*q* = 0, consistent with the undisplaced limit in which all FC intensity collapses into the zero-phonon line and the only vibronic sidebands are HT-driven, so absorption and emission must be equal. This shared value at the origin corresponds to ζ = 0, as implied by eq 10.

**Figure 3:**
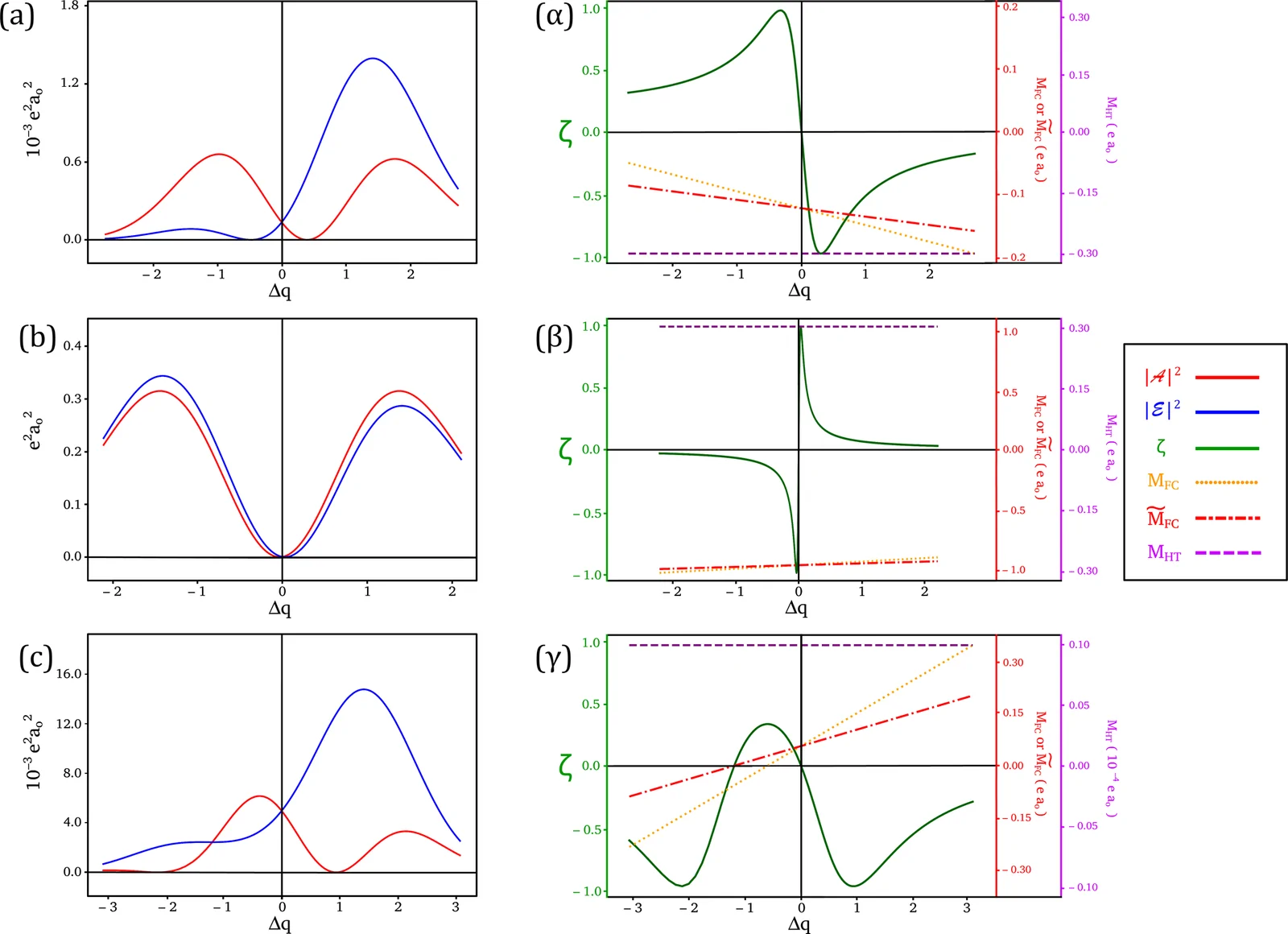
Representative single-mode FC–HT asymmetry scans for totally symmetric HT-active CSPs. Left panels show the absorption $|A{|}^{2}$ and emission $|E{|}^{2}$ transition-dipole strengths as functions of the dimensionless displacement Δ*q*. Right panels show the corresponding asymmetry factor ζ (green, left axis), together with M_FC_ (orange dotted), M̃_FC_ (red dash-dot), and M_HT_ (purple dashed) on the right axis. Panels (a)/(α): Phenanthrene 4­(*a*_1_)^0→1^ (in-plane ring deformation), *z* axis, no sign change in M̃_FC_. Panels (b)/(β): Anthracene 4­(a_g_)^0→1^ (in-plane C–H bending mode), *x* axis, no sign change in M̃_FC_. Panels (c)/(γ): Pyrene 3­(a_g_)^0→1^ (skeletal breathing), *y* axis, sign change in M̃_FC_.

Away from the origin, both curves develop a characteristic double-peaked envelope. This follows from the closed-form FC overlap of eq 11, where the polynomial factor (Δ*q*)^Δν^ grows away from the origin while the Gaussian envelope exp­(−Δ*q* ^2/4^) decays at large displacements, producing a maximum at intermediate |Δ*q*| on each side. For higher-order Laguerre polynomials, additional nodes and subsidiary maxima appear, but the overall double-peaked character is preserved.

In all cases, ζ changes sign as Δ*q* crosses zero. As required by eq 32, the direction of the FC–HT interference follows the direction of the distortion. In the more familiar parity language, F^(u;w)^ and H^(u;w)^ must carry opposite parity in Δ*q* for any given Δν, so that their product (the interference term) is always odd. The observed reversal of ζ at Δ*q* = 0 is thus invariant with respect to the specific vibronic levels involved and is ultimately another manifestation of harmonic-oscillator parity, the same symmetry principle that underlies FC–HT interference in the first place.

This reversal is the clearest demonstration that the signed displacement, and not the Huang–Rhys factor, is the appropriate variable for describing FC–HT interference. The Huang–Rhys factor is precisely the argument $\alpha =\frac{{\Delta q}^{2}}{2}$ of the Laguerre polynomial in eq 11 and is even in Δ*q* by construction. The Laguerre and Gaussian factors therefore depend on the displacement only through α, and all sign information resides in the prefactor (Δ*q*)^Δν^ and, equivalently, in γ.

Beyond the sign change at the origin, panel (γ) illustrates the role of the electronic prefactors contained in eq 32. In the fixed-reference linear-HT treatment, M_HT_ is evaluated at the reference geometry and remains constant throughout the displacement scan, while the midpoint-shifted FC prefactor M̃_FC_ varies linearly with Δ*q*. Consequently, additional sign changes in ζ can occur only where M̃_FC_ passes through zero. Together with the reversal at Δ*q* = 0 and the Δν dependence discussed in Section 4.1, these results recover the sign behavior predicted by eq 32 within a single globally linear HT expansion.

Since M_HT_ and Δ*q* change sign together under a redefinition of the coordinate, eq 32 can be restated without reference to any sign convention. The invariant content is the sign of M̃_FC_ M_HT_Δ*q*, which is negative precisely when |M­(*q*)| decreases upon passing from the ground-state minimum to the excited-state minimum. For Δν > 0, the branch of the scan on which |M­(*q*)| decreases, therefore, corresponds to ζ > 0, and the branch on which it increases corresponds to ζ < 0; for Δν < 0, the assignment is reversed.

The reduction of the asymmetry factor to a single dependence on κ in eq 20 makes the shape of the asymmetry curve straightforward to account for. In what follows, a *node* denotes a displacement at which ζ vanishes, that is, at which one of the two interference pathways closes. Nodes are of two kinds: (i) poles, where |κ| → ∞, arising at Δ*q* = 0 or wherever M̃_FC_ vanishes; and (ii) zeros, where κ → 0, arising as |Δ*q*| → ∞.

Between nodes there lies extremal behavior. Using eq 16 and eq 9, the two members of a CSP factorize as

33 $$A=F^{\left(\mathrm{u;w}\right)}\left({\mathrm{M̃}}_{\mathrm{FC}}+M_{\mathrm{HT}}\gamma \right),,E=\sigma F^{\left(\mathrm{u;w}\right)}\left({\mathrm{M̃}}_{\mathrm{FC}}-M_{\mathrm{HT}}\gamma \right)$$

so that complete asymmetry, |ζ| = 1, is reached when |M_HT_γ| = |M̃_FC_|. At such a displacement, the FC and HT amplitudes of one member cancel identically, and its vibronic line is extinguished. Because |κ| is continuous between nodes, the number of such extinctions is constrained by the character of the nodes bounding the interval. Between adjacent nodes of opposite kind, |κ| must pass from above unity to below it, and complete extinction therefore occurs an odd number of times, at least once. Between adjacent nodes of the same kind, the crossings are even in number and may be absent altogether. Complete extinction of one member of a CSP is, in this sense, a generic feature of the displaced-oscillator limit rather than a coincidence of particular parameters.

The displacement scan is, of course, a theoretical construction, since a given molecule usually presents only a single Δ*q* per mode. The structure identified here is nonetheless observable in a single spectrum; how this is achieved is discussed in detail in Section 4.2.3.

#### 4.2.2 Nondegenerate Nontotally Symmetric HT-Active Modes

It is often assumed that FC–HT interference is irrelevant for nontotally symmetric promoting modes. The usual argument rests on symmetry selection rules at a high-symmetry reference geometry. For a nontotally symmetric mode, the FC integral is nonzero only for even changes in vibrational quanta, Δν = 0, ±2, ±4, ±6, ..., whereas the HT integral is nonzero only for odd changes, Δν = ±1, ±3, ±5, ..., reducing further to the strict Δν = ±1 rule in the equal-frequency harmonic limit.^30^

A second, electronic selection rule operates alongside this. For a nontotally symmetric mode, symmetry places its corresponding M_HT_ on a component of the transition dipole orthogonal to the FC-allowed one. On the FC-allowed component, this mode therefore contributes no HT term (any HT activity on that component arises from totally symmetric modes and is the subject of Section 4.2.1). The analysis below concerns the FC-forbidden component on which M_HT_ lies, hereafter the borrowed component, where M_FC_ vanishes exactly at Δ*q* = 0 and where intensity borrowing occurs.

Crucially, however, these selection rules presume that both electronic states retain the same point group, which forces the minima along any nontotally symmetric coordinate to coincide,^22^ meaning Δ*q* = 0. If the electronic transition lowers the symmetry, Δ*q* for a nontotally symmetric mode can be nonzero. Because displacement along a nontotally symmetric normal coordinate inherently lowers the local point group symmetry, the strict symmetry-based selection rules separating even and odd Δν channels break down when Δ*q* ≠ 0. The electronic rule relaxes with them, meaning the borrowed component that originally only carried HT intensity at the parent geometry can gain a nonzero M_FC_ value. Consequently, both FC and HT contributions can become simultaneously nonzero for a given transition on the same component, meaning that FC–HT interference is no longer strictly forbidden for nontotally symmetric modes. It should be stressed that this mechanism requires no dependence of the transition dipole on terms beyond first order.

In contrast to a totally symmetric coordinate, for a nondegenerate, nontotally symmetric coordinate the distortion preserves only the kernel of the parent point group^20,21^ and the associated one-dimensional deformation space must contain at least one nontrivial symmetry operation that identifies +Δ*q* with −Δ*q*. Only the magnitude of the displacement is, therefore, physically meaningful, and the scans in Figure 4 are plotted against |Δ*q*|. As such, while the sign behavior of the asymmetry curve is still described by eq 32, Δ*q* here must always be positive in value.

**Figure 4:**
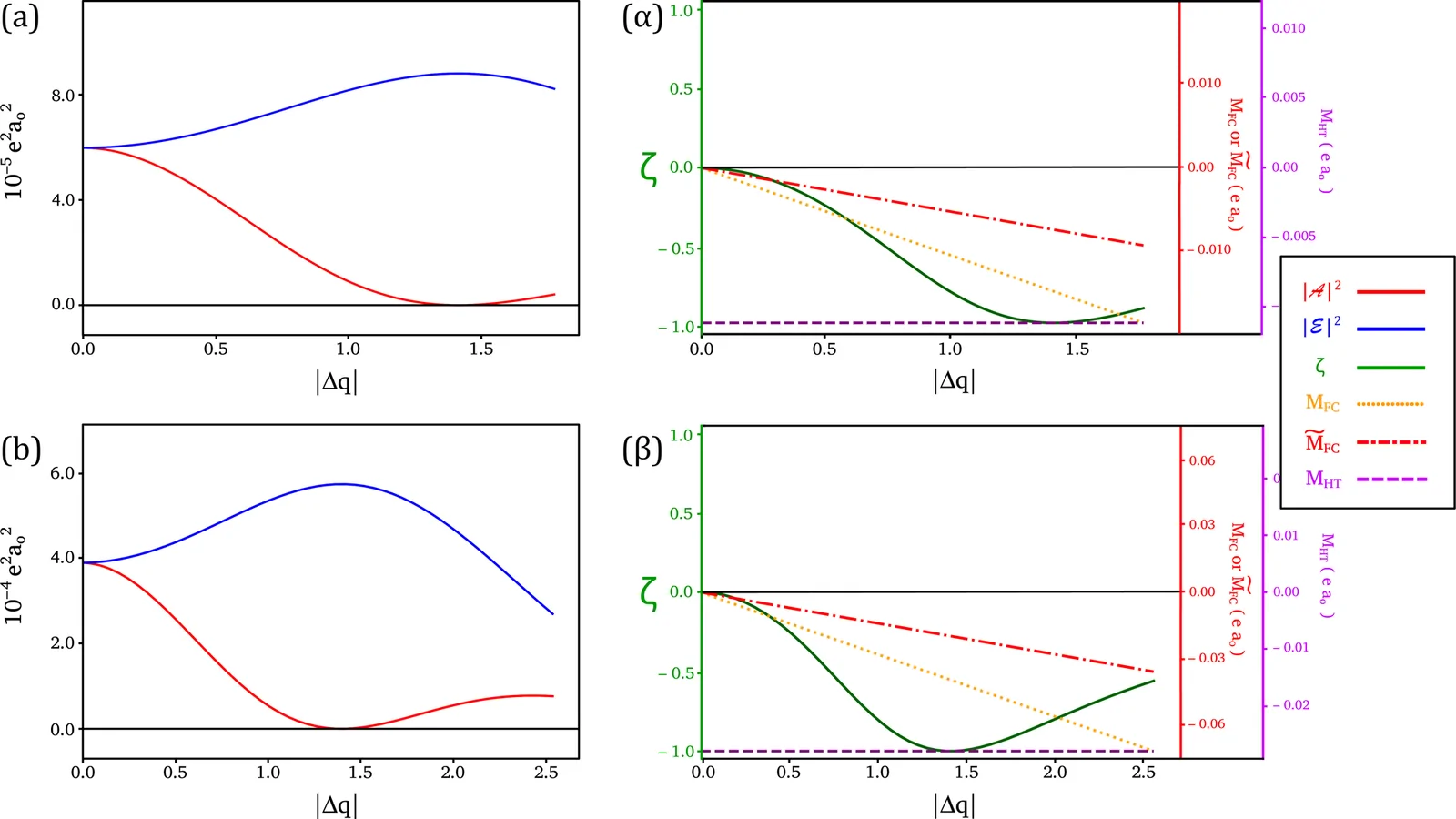
Representative single-mode FC–HT asymmetry scans for nondegenerate nontotally symmetric HT-active CSPs. Left panels show the absorption $|A{|}^{2}$ and emission $|E{|}^{2}$ transition-dipole strengths as functions of the dimensionless displacement Δ*q*. Right panels show the corresponding asymmetry factor ζ (green, left axis), together with M_FC_ (orange dotted), M̃_FC_ (red dash-dot), and M_HT_ (purple dashed) on the right axis. Since +Δ*q* and −Δ*q* are symmetry-equivalent for these modes, only |Δ*q*| is shown. In each case the quantities plotted are those of the borrowed component, that is, the component on which the M_HT_ of the scanned mode lies and for which the transition is FC-forbidden at the reference geometry, so that M_FC_ = 0 at Δ*q* = 0. Panels (a)/(α): Formaldehyde 1­(b_2_)^0→1^ (in-plane CH_2_ rock), *x* component. Panels (b)/(β): (*E*)-difluoroethene 4­(b_u_)^0→1^ (antisymmetric C–H stretch), *z* component. The two ζ curves are identical despite having different electronic prefactor values.

The qualitative difference from the totally symmetric case lies in the behavior near Δ*q* = 0. On the borrowed component, M_FC_ vanishes exactly at the reference geometry, so both members of the CSP are HT-only there and coincide, giving ζ = 0. Displacing along the inducing coordinate relaxes the selection rule as described above, so the transition dipole on that component is directly proportional to M_HT_Δ*q*, and the midpoint-shifted FC prefactor becomes ${\mathrm{M̃}}_{\mathrm{FC}}=\frac{1}{2}M_{\mathrm{HT}}\Delta q$ .

The electronic prefactors are therefore not independent in this case, and two equivalent consequences follow. First, the transition-dipole magnitude |M­(*q*)| must increase away from the origin. Second, because M_FC_ can leave zero only in a definite direction, M̃_FC_ and M_HT_ are locked to the same relative sign initially.

The electronic ratio entering the reduced variable is therefore fixed by the displacement alone, MHT/M̃_FC_ = 2/Δ*q*, and eq 19 collapses to

34 $$\kappa =-\frac{2\Delta \nu }{{\Delta q}^{2}}$$

Every electronic quantity cancels out of this expression. Within the fixed-reference linear HT model, the asymmetry of a nontotally symmetric mode is therefore determined only by the vibrational quantum change and the magnitude of the displacement. The sign rule follows immediately, since ζ carries the sign of κ:

35 sgn(ζ)=−sgn(Δν)

Equation 34 also fixes the position of complete asymmetry. Since |ζ| = 1 when |κ| = 1, the extrema occur at

36 $${\Delta q}^{*}=\sqrt{2\left|\Delta \nu \right|}$$

The extremum position is independent of the magnitude or sign of M_HT_ and depends only on |Δν|. For a fundamental transition with |Δν| = 1, the extrema therefore occur at $\left|\Delta q^{*}\right|=\sqrt{2}≃1.41$ . This differs from the totally symmetric case discussed in Section 4.2.1, where the reference FC contribution remains nonzero and the electronic ratio is an independent parameter.

The two panels of Figure 4 illustrate this parameter-free behavior for two molecular examples. Although the underlying HT prefactors, and thus the transition-dipole strength curves, differ between the two systems, their asymmetry curves follow exactly the same dependence on Δ*q* and Δν.

This universal behavior provides a useful benchmark for assessing finite-displacement electronic effects. In the geometry-reparametrized calculations presented in the Supporting Information, the transition dipole and its derivative are reevaluated along the displacement coordinate. Departures from eq 34, including shifts in the position of the asymmetry extrema, therefore measure the geometry dependence introduced beyond the fixed-reference linear expansion. It should be noted that, because the recomputed prefactors are evaluated at the actual distorted geometry, these departures carry both the nonlinear variation of the electronic transition moment along the coordinate and the effect of the finite rectilinear displacement itself, which at large amplitudes no longer corresponds to motion along a single normal coordinate. The present comparison does not separate the two.

#### 4.2.3 Vibronic State

Two distinct dependencies on the vibronic state are isolated in Figure 5. The top row (panels a and α) follows *p*-dichlorobenzene displaced along 1­(a_g_) for the CSPs 1­(a_g_)^0→1^, 1­(a_g_)^1→2^, and 1­(a_g_)^2→3^, which share the spacing Δν = 1 but differ in absolute vibronic level. The bottom row (panels b and β) follows anthracene displaced along 9­(a_g_) for the CSPs 9­(a_g_)^0→1^, 9­(a_g_)^0→2^, and 9­(a_g_)^0→3^, which share the initial level *u* = 0 but differ in spacing.

**Figure 5:**
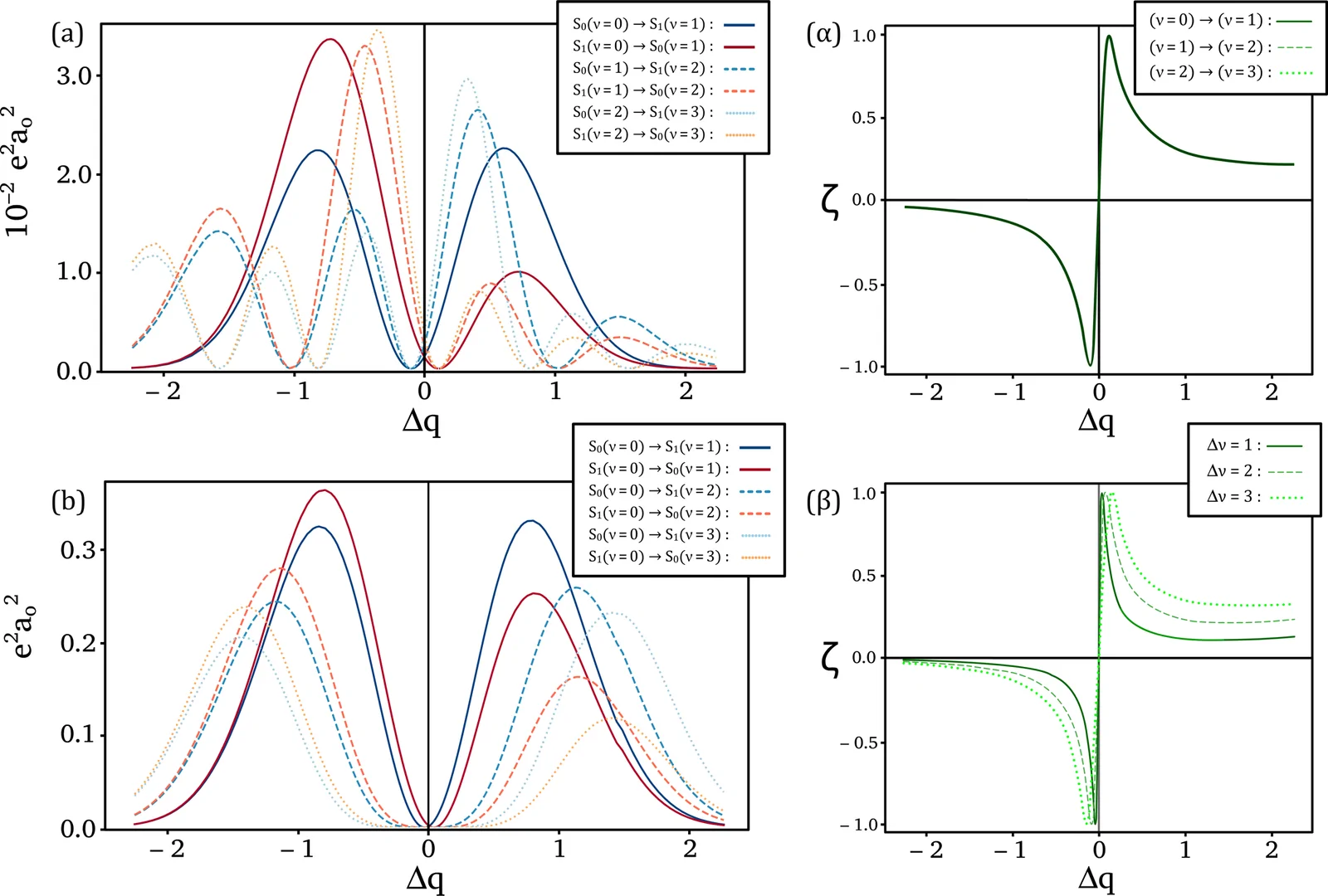
Effect of the vibronic state on single-mode FC–HT interference. The top row, panels (a) and (α), shows *p*-dichlorobenzene displaced along normal mode 1­(a_g_) (symmetric C–Cl stretch) for the CSPs 1­(a_g_)^0→1^, 1­(a_g_)^1→2^, and 1­(a_g_)^2→3^, which share the vibrational spacing Δν = 1 but differ in absolute vibronic level. The bottom row, panels (b) and (β), shows anthracene displaced along normal mode 9­(a_g_) (aromatic C–C ring stretch) for the CSPs 9­(a_g_)^0→1^, 9­(a_g_)^0→2^, and 9­(a_g_)^0→3^, which share the initial level *u* = 0 but differ in spacing Δν = w – u. The left panels (a, b) show the transition-dipole strengths in absorption and in the corresponding mirror emission lines, and the right panels (α, β) show the associated asymmetry factors ζ­(Δ*q*). Although the absolute dipole strengths depend on the specific vibronic levels, the asymmetry curves coincide for all transitions with the same vibrational spacing, as in panel (α). Increasing |Δν| suppresses intensity near Δ*q* = 0 and shifts the dominant maxima in both the dipole strengths and ζ­(Δ*q*) toward larger |Δ*q*| while retaining a similar overall curve shape, as in panels (b) and (β).

Panel (a) shows that the absolute transition dipole strengths differ for u → w transitions that share the same spacing Δν = w – u. This is expected from eq 11, whose magnitude depends explicitly on *u* through both the factorial prefactor and the degree of the associated Laguerre polynomial. Because the HT integral is built from neighboring FC overlaps, it inherits the same dependence on the absolute vibronic levels.

By contrast, panel (α) shows that the asymmetry factor ζ­(Δ*q*) is invariant with respect to the absolute values of *u* and *w* and depends only on Δν, so the three curves collapse into one. This is a direct result of eq 16, where γ depends only on the spacing and displacement, and the common factor (F^(u;w)^)^2^ carrying all the *u* dependence cancels between the numerator and denominator of eq 10. The three CSPs of panel (α) are compared at the same displacement and therefore at identical electronic prefactors, so the collapse is exact, and the differing absolute strengths of panel (a) are entirely absorbed in the normalization.

The lower row isolates the remaining dependence, namely that on Δν itself. Panel (b) shows that as |Δν| increases, the maxima of the transition dipole strengths shift to larger |Δ*q*|. This follows from eq 11, in which the dominant Δν dependence, at fixed *u,* enters through the factor (Δ*q*)^Δν^ that multiplies the Gaussian envelope exp­(−Δ*q* ^2^/4). Raising the order of this polynomial suppresses the overlap near Δ*q* = 0 and pushes the intensity maximum outward. The overall shape of each curve is nevertheless largely preserved, since for a given initial state *u* the detailed modulation of the overlap is set by the associated Laguerre polynomial, so changing Δν mainly relocates the dominant maximum rather than introducing new structure.

Panel (β) shows how the asymmetry changes with Δν. At any fixed displacement, the electronic quantities M_HT_ and M̃_FC_ are the same for all CSPs, so eq 19 gives κ ∝ Δν. Increasing |Δν| therefore moves the system through the universal dependence of ζ on κ. As |Δν| increases, the extrema in the displacement scans shift outward, as observed in panel (β). This shift is not, however, a simple linear stretching of the displacement axis because M̃_FC_ itself varies linearly with Δ*q* in the fixed-reference treatment. The positions of the extrema therefore reflect the combined dependence of κ on Δν, Δ*q*, and the midpoint-shifted FC prefactor.

A displacement scan is a theoretical construction, since a given molecule presents a single Δ*q* per mode. What a single spectrum obtained from experiment does supply is a range of Δν at fixed geometry, and hence at genuinely fixed electronic prefactors. Writing the asymmetry factor as a function of Δν instead turns eq 20 into

37 $$\zeta \left(\Delta \nu \right)=\frac{2\tau \Delta \nu }{1+\tau^{2}{\Delta \nu }^{2}},,\tau =-\frac{M_{\mathrm{HT}}}{{\mathrm{M̃}}_{\mathrm{FC}}\Delta q}$$

so that a vibronic progression samples the universal curve at fixed geometry in the same way that a scan samples it at fixed Δν. The progression is a vertical cut through panel (β), rather than one of its curves. According to eq 37, |ζ| is unimodal in |Δν|, with a single maximum at Δν* = |τ|^–1^. The asymmetry therefore grows with the number of quanta for |Δν| < Δν* and falls beyond it, and the direction of the trend across an observed progression fixes which regime the band occupies.

Examining the asymmetry trend of a vibronic progression thus resolves an ambiguity that a single cross-swap pair cannot. Equation 20 is invariant under κ → κ^–1^, so a measured ζ at one Δν is reproduced by two values of τ, one below and one above |Δν|^–1^, corresponding to weak HT coupling at larger displacement and to strong coupling at smaller displacement. An isolated line, therefore, fixes the magnitude of the asymmetry but not the regime that produced it. The two candidates predict distinct behavior across the progression, so that measurements at additional values of Δν can select between them and thereby determine the relevant branch of τ, and with it the ratio of the electronic prefactors to the displacement. The same logic extends beyond a single progression. Each progression returns only a ratio, but M_FC_ is common to the modes of a given band, so progressions of different modes are read against a shared denominator. Given displacements from the Huang–Rhys factors of the same spectrum, this offers a route to the relative HT prefactors across modes.

#### 4.2.4 Experimental Illustration: Gas-Phase Phenanthrene

A particularly simple experimental example to demonstrate the asymmetry analysis detailed in Section 4.2.3 is provided by the jet-cooled S_1_ ↔ S_0_ spectrum of phenanthrene reported by Warren, Hayes, and Small.^31^ Photoexcitation and dispersed-fluorescence spectra were measured for isolated phenanthrene in a supersonic expansion of helium. For the low-frequency, totally symmetric *a*_1_ modes, little evidence was found for Duschinsky mixing or Fermi resonance, while the observed overtone and combination-band anharmonicities were typically only 0–2 cm^–1^. The pronounced absorption–fluorescence asymmetry of these modes was therefore assigned primarily to interference between Condon and first-order Herzberg–Teller contributions. This places the experiment close to the displaced-harmonic limit for which eq 37 was derived.

Modes 20­(a_1_), 21­(a_1_), and 22­(a_1_) are especially useful because Warren et al. reported fundamental intensities along with first-overtone intensities or experimental bounds. The experimental data and the corresponding progression analysis are presented in Table 1. Within each photoexcitation or fluorescence spectrum, the reported intensities are normalized to the corresponding Δν = 0 transition. The mass-weighted displacements and HT derivatives reported in ref 31 have also been converted in Table 1 to the dimensionless normal-coordinate convention used here.

**Table 1:** Gas-Phase Phenanthrene Data from Warren et al.^31^ and the Corresponding Progression Analysis^a^

	experimental input							progression analysis						
mode	ω_S_1__	ω_S_0__	*I*_A_^exp^(1)	*I*_E_^exp^(1)	*I*_A_^exp^(2)	*I*_E_^exp^(2)	Δ*q*	ζ_1_^exp^	ζ_2_^exp^	τ_<_^(1)^	τ_>_^(1)^	τ_prog_	${|\frac{M_{\mathrm{HT},X}}{M_{\mathrm{HT},20(a_{1})}}|}_{\mathrm{prog}}$	${|\frac{M_{\mathrm{HT},X}}{M_{\mathrm{HT},20(a_{1})}}|}_{W}$
20(a_1_)	673	714	84	12	7	<5	0.365	0.750	>0.167	0.451	2.215	–	1.000^†^	1.000
21(a_1_)	517	544	43	6	1	<1	0.276	0.755	>0	0.456	2.193	–	0.749^†^	0.756
22(a_1_)	395	408	0.3	21	0.2	1	0.276	–0.972	–0.667	–0.786	–1.271	–1.30	0.434	0.457

a The experimental photoexcitation and dispersed-fluorescence intensities, *I*_A_^exp^ and *I*_E_^exp^, are normalized within each spectrum to the corresponding Δ*ν* = 0 transition, set to 100. The mass-weighted equilibrium displacements δ_*x*_ reported in ref 31 have been converted to the dimensionless normal-coordinate displacements Δ*q* used in the present work using the corresponding S_0_ vibrational frequencies. The experimental asymmetries were obtained from eq 38. The quantities τ_<_^(1)^ and τ_>_^(1)^ are the two reciprocal solutions obtained from the fundamental (Δ*ν* = 1) alone, with |τ_<_^(1)^| < 1 and |τ_>_^(1)^| > 1. *τ*_prog_ denotes the value obtained when the measured overtone also permits the progression to be fitted; this is possible for 22­(a_1_) but not for 20­(a_1_) or 21­(a_1_) because only upper bounds were reported for their fluorescence overtones. The final two columns compare the relative HT prefactors, normalized to mode 20­(a_1_). The progression-derived values were obtained from eq 41 with Y = 20­(a_1_); for modes marked †, the large-|τ| fundamental branch is used because the progression itself does not resolve the two branches. The Warren values were obtained by converting the reported mass-weighted *m*_*x*_/*M*_0_ parameters to the dimensionless-coordinate convention and normalizing the resulting |M_HT_| values to mode 20­(a_1_). Only magnitudes are compared because the phase of each normal coordinate is arbitrary.

The analysis proceeds directly from the measured absorption–emission asymmetry. For each member *n* of a progression, we define

38 $$\zeta_{n}^{\exp}=\frac{I_{A}^{\exp}\left(n\right)-I_{E}^{\exp}\left(n\right)}{I_{A}^{\exp}\left(n\right)+I_{E}^{\exp}\left(n\right)}$$

The fundamental, Δν = 1, then fixes the magnitude of the interference but does not by itself determine on which side of the universal curve the transition lies. Inverting eq 37 gives the two reciprocal solutions

39 $$\tau_{\pm }=\frac{1\pm \sqrt{1-{\left(\zeta_{1}^{\exp}\right)}^{2}}}{\zeta_{1}^{\exp}}$$

listed in Table 1 as τ_<_^(1)^ and τ_>_^(1)^, with |τ_<_^(1)^| < 1 and |τ_>_^(1)^| > 1. A second member of the same progression therefore provides the additional information required to discriminate between the two branches.

This distinction is seen directly in the three phenanthrene modes. For 20­(a_1_) and 21­(a_1_), the fluorescence overtones were reported only as upper bounds. Their measured progressions therefore constrain the sign and magnitude of the asymmetry but do not uniquely select between the reciprocal τ solutions. Mode 22­(a_1_) presents a more restrictive case. Its fundamental gives the two possibilities τ = −0.786 and −1.271, while the measured overtone asymmetry is consistent with the large-|τ| branch and inconsistent with the small-|τ| alternative. Fitting the fundamental and overtone simultaneously refines this value to τ_prog_ = −1.30. The important point is therefore not the numerical inversion itself but that the progression resolves information that is fundamentally degenerate for an isolated cross-swap pair.

The comparison should be regarded as semiquantitative, because the experimental quantities are relative photoexcitation and fluorescence intensities rather than frequency-corrected transition strengths. Nevertheless, the small changes in vibrational frequency, weak anharmonicity, and the absence of appreciable low-frequency a_1_ mode mixing make phenanthrene a useful experimental illustration of the progression dependence predicted by eq 37.

The same analysis also permits the relative HT coupling strengths of different modes to be compared. For modes belonging to the same electronic band,

40 $$\tau_{X}=-\frac{M_{\mathrm{HT},X}}{{\mathrm{M̃}}_{\mathrm{FC}}{\Delta q}_{X}}$$

so that the common electronic denominator cancels in a mode-to-mode ratio,

41 $$\frac{\left|M_{\mathrm{HT},X}\right|}{\left|M_{\mathrm{HT},Y}\right|}=\frac{\left|\tau_{X}{\Delta q}_{X}\right|}{\left|\tau_{Y}{\Delta q}_{Y}\right|}$$

Thus, once the displacements are known, the progression-derived τ_X_ values determine the relative HT prefactors without requiring the absolute value of the Condon transition moment.

For mode 22­(a_1_), the large-|τ| branch is selected directly by the measured progression. For 20­(a_1_) and 21­(a_1_), the overtone bounds do not independently resolve the ambiguity, so the large-|τ| branches consistent with the first-order parametrization of Warren et al. are used for the cross-mode comparison. As shown in the final two columns of Table 1, the relative HT magnitudes inferred from the progression analysis closely reproduce those obtained by converting the fitted mass-weighted HT derivatives of Warren et al. to the same dimensionless-coordinate convention, differing by only 0.9% for mode 21­(a_1_) and 5.0% for mode 22­(a_1_) relative to the Warren values.

This close correspondence provides a useful consistency check on the reduced description: the progression-based analysis recovers essentially the same mode-to-mode variation of the HT coupling strength as the explicit first-order fit, while requiring only the measured asymmetry, the displacement, and the common electronic denominator. This comparison is not an independent validation of the Warren parametrization, since those displacements and HT derivatives were themselves obtained by fitting the same vibronic intensity data. Rather, it demonstrates that the universal progression variable provides an equivalent and considerably reduced route to the mode-to-mode HT information. Because the phase of each normal coordinate is arbitrary, only the relative HT magnitudes are compared.

### 4.3 Combination Transitions

This section considers combination bands involving only totally symmetric HT-active modes, specifically, transitions in which two totally symmetric normal coordinates are simultaneously excited. Figure 6 shows a representative asymmetry map, ζ­(Δ*q*_A_, Δ*q*_B_), for a two-mode combination transition as a function of the dimensionless displacements along modes A and B. Any one-dimensional cut through this surface (fixing Δ*q*_B_ and scanning Δ*q*_A_, or vice versa) retains the characteristic nodal and extremal structure of the single-mode case, while carrying an additional fixed contribution from the second HT pathway.

**Figure 6:**
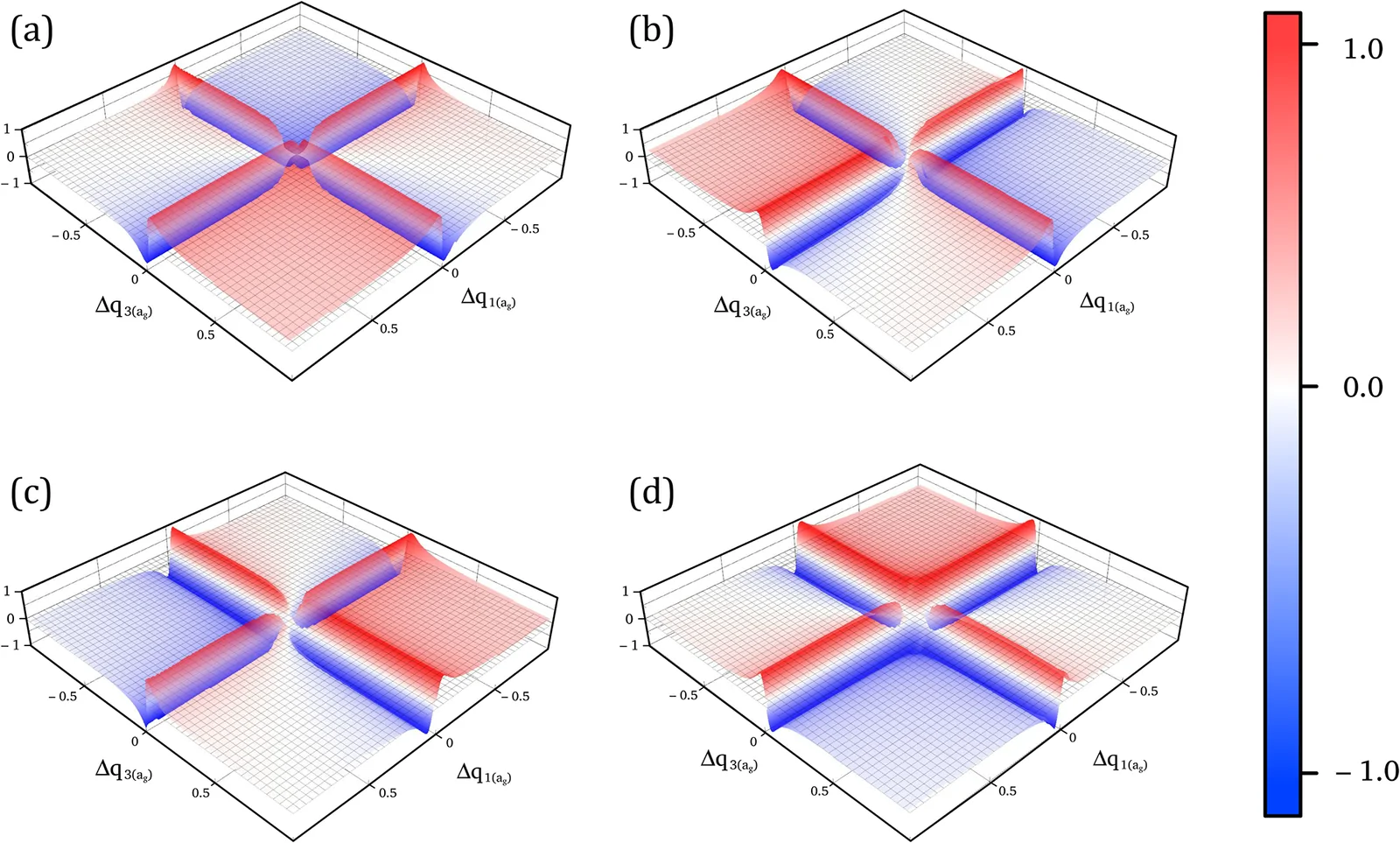
Asymmetry-factor surfaces $\zeta \left(\Delta q_{1(a_{g})},\Delta q_{3(a_{g})}\right)$ for the *x*-polarized combination band in ethene involving modes 1­(a_g_) (symmetric CH_2_ scissor) and 3­(a_g_) (symmetric C–H stretch), where the midpoint-shifted FC prefactor does not change sign within the scanned range and the two fixed-reference HT prefactors are nonzero. Panels correspond to different choices of vibrational direction for each mode: (a) 1­(a_g_)^0→1^ 3­(a_g_)^0→1^, (b) 1­(a_g_)^0→1^ 3­(a_g_)^1→0^, (c) 1­(a_g_)^1→0^ 3­(a_g_)^0→1^, and (d) 1­(a_g_)^1→0^ 3­(a_g_)^1→0^. In all cases, nodal lines occur along $\Delta q_{1(a_{g})}=0$ and ${\Delta q}_{3(a_{g})}=0$ , reflecting the parity-imposed sign reversal when either displacement changes sign. A further diagonal nodal line crossing the origin appears within two of the quadrants, arising from cancellation between the two mode-specific HT pathways in the net interference.

A two-mode combination band carries two distinct first-order HT pathways. In the separable displaced-oscillator limit the corresponding overlaps factorize as

42 $$H_{A}^{\left(u_{A}u_{B};w_{A}w_{B}\right)}=\left\langle \chi_{\mathrm{GS}:u_{A}u_{B}}\left|q_{M,A}\right|\chi_{\mathrm{ES}:w_{A}w_{B}}\right\rangle =H_{A}F_{B}$$

43 $$H_{B}^{\left(u_{A}u_{B};w_{A}w_{B}\right)}=\left\langle \chi_{\mathrm{GS}:u_{A}u_{B}}\left|q_{M,B}\right|\chi_{\mathrm{ES}:w_{A}w_{B}}\right\rangle =H_{B}F_{A}$$

so that the HT correction entering the interference is a signed sum of two pathway contributions rather than a single-mode quantity. Expanding the transition dipole about the excited-state geometry and converting each coordinate to its midpoint form,

44 $$M\left(q\right)={\mathrm{M̃}}_{\mathrm{FC}}+M_{\mathrm{HT},A}q_{M,A}+M_{\mathrm{HT},B}q_{M,B}$$

45 $${\mathrm{M̃}}_{\mathrm{FC}}=M_{\mathrm{FC}}-\frac{{\Delta q}_{A}}{2}M_{\mathrm{HT},A}-\frac{{\Delta q}_{B}}{2}M_{\mathrm{HT},B}$$

so the shifted FC prefactor now collects a contribution from each displaced mode. It nevertheless remains a single scalar common to both HT pathways, since both borrow from the same electronic transition, and the reduction of Section 2.2 therefore carries over unchanged. Applying eq 16 to each mode separately gives

46 $$A=F_{A}F_{B}\left({\mathrm{M̃}}_{\mathrm{FC}}+M_{\mathrm{HT},A}\gamma_{A}+M_{\mathrm{HT},B}\gamma_{B}\right)$$

47 $$E=\sigma_{A}\sigma_{B}F_{A}F_{B}\left({\mathrm{M̃}}_{\mathrm{FC}}-M_{\mathrm{HT},A}\gamma_{A}-M_{\mathrm{HT},B}\gamma_{B}\right)$$

The common factor F_A_F_B_ and the parity factors cancel in the normalized asymmetry, and the combination band therefore obeys the same universal form as the single-mode case, eq 20, with the reduced variable generalized to a sum over pathways,

48 $$\kappa =\frac{M_{\mathrm{HT},A}\gamma_{A}+M_{\mathrm{HT},B}\gamma_{B}}{{\mathrm{M̃}}_{\mathrm{FC}}}$$

The entire two-dimensional map is thus fixed by a single scalar field κ­(Δ*q*_A_, Δ*q*_B_), and every feature of Figure 6 follows from where this field vanishes, diverges, or passes through unity.

Two kinds of node are visible, corresponding respectively to poles and zeros of κ. The first are poles of κ: the axial nodes at Δ*q*_A_ = 0 and Δ*q*_B_ = 0, where the corresponding γ_X_ diverges and hence ζ → 0. These are the two-dimensional counterparts of the origin node of the single-mode scans, and a one-dimensional cut through the surface at fixed Δ*q*_B_ reproduces the familiar single-mode behavior across the Δ*q*_A_ = 0 node, and vice versa. The second is a zero of κ, arising where the two HT pathways cancel, M_HT,A_γ_A_ = −M_HT,B_γ_B_, which happens along

49 $$\frac{{\Delta q}_{B}}{{\Delta q}_{A}}=-\frac{M_{\mathrm{HT},B}{\Delta \nu }_{B}}{M_{\mathrm{HT},A}{\Delta \nu }_{A}}$$

a straight line through the origin whose slope is fixed by the ratio of the HT electronic prefactors weighted by the vibrational quantum changes. This diagonal node is a genuinely multidimensional feature with no single-mode analogue, since it exists only because two pathways contribute to the same vibronic line. Because |κ| must pass from one limit to the other between an axial node and the diagonal node, the contour |κ| = 1 lies between them, and these are the ridges of complete asymmetry visible in Figure 6.

Equation 48 also fixes the sign structure of the map, taking the sign of κ and clearing the denominators of γ_A_ and γ_B_,

50 $$\mathrm{sgn}\left(\zeta \right)=-\mathrm{sgn}\left({\Delta q}_{A}{\Delta q}_{B}\right)\;\mathrm{sgn}\left({\mathrm{M̃}}_{\mathrm{FC}}\left(M_{\mathrm{HT},A}{\Delta q}_{B}{\Delta \nu }_{A}+M_{\mathrm{HT},B}{\Delta q}_{A}{\Delta \nu }_{B}\right)\right)$$

which reduces to eq 32 when either mode is inactive. A useful way to read the resulting map is to partition the (Δ*q*_A_, Δ*q*_B_) plane into four sign quadrants. For a fixed Δν branch each mode has a preferred displacement sign that yields ζ  > 0, and in the quadrant where both displacements carry their single-mode ζ  >  0 signs the two HT terms in eq 48 share a sign, reinforce, and the quadrant is uniformly positive. The opposite quadrant is uniformly negative for the same reason. In the two remaining quadrants the pathways enter with opposite signs and compete, so eq 49 is satisfied somewhere within each, and the diagonal node divides the quadrant into subregions of opposite ζ.

The two quantum-number changes act independently, which has no single-mode counterpart. In the single-mode case, reversing Δν reverses the entire asymmetry curve. Here, each Δν_X_ enters only through its own γ_X_, so flipping Δν_A_ reverses the contribution of mode A while leaving that of mode B unchanged. This is a selective reversal along one coordinate rather than a global sign change, and it also reflects the diagonal node of eq 49 about the vertical axis. Viewed on the (Δ*q*_A_, Δ*q*_B_) plane, the quadrant assignments are reshuffled, appearing as a 90° rotation of the asymmetry surface, as in panels (b) and (c) of Figure 6. Flipping both quantum-number changes reverses both contributions, recovering the original orientation with the sign reversed everywhere, a 180^◦^ rotation, as in panel (d).

For combination transitions involving three or more modes, the same structure persists. Each additional HT-active mode contributes a further term M_HT,X_γ_X_ to the numerator of eq 48, so the asymmetry remains a function of the single scalar κ, and the cancellation condition $\underset{X}{\sum }M_{\mathrm{HT},X}\gamma_{X}=0$ defines a nodal hypersurface of codimension one in the space of displacements.

### 4.4 Extension to Duschinsky-Mixed Modes

The analysis so far has deliberately been restricted to the AS limit in which the ground- and excited-state harmonic potentials share a common normal-mode basis. In this limit, the Duschinsky matrix is the identity, and the multidimensional vibrational overlap integrals factorize into products of one-dimensional displaced-oscillator overlaps. In real polyatomic molecules, however, the normal coordinates of the two electronic states do not necessarily coincide. Instead, an excited-state normal mode may be a linear combination of several ground-state normal modes with the same symmetry.

Figure 7 provides a direct spectral-level illustration of the effect of Duschinsky mixing. As can be seen, transitions that are mirror-symmetric in the AS limit (θ = 0) can become asymmetric once mode mixing is introduced. Reversing the sign of the mixing angle between panels also reverses the direction of the asymmetry for several, though not all, classes of transitions.

**Figure 7:**
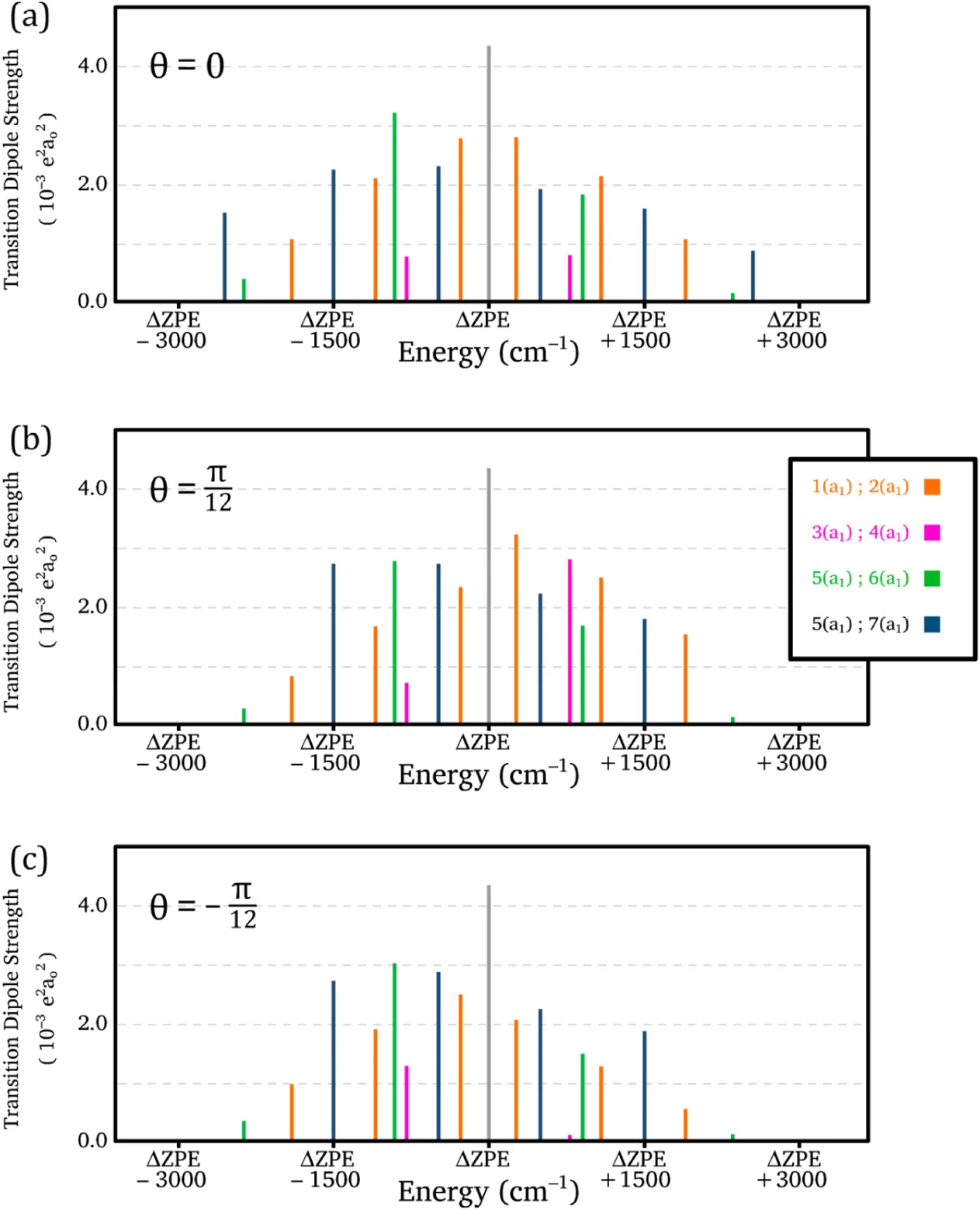
Simplified simulated stick spectra showing the effect of Duschinsky mixing on absorption–emission asymmetry, computed at the FC–HT level for four two-mode combination bands of pyridazine. Each color denotes one two-mode pair, with absorption and emission drawn in the same color and distinguished by their position relative to the zero-phonon line. Within each pair, the first-named mode is the participant, whose vibrational quanta change across the transition, and the second is the partner, held at Δν = 0 throughout. Panels show (a) the adiabatic-shift limit θ = 0, and the same bands after mixing through (b) $\theta =+\frac{\pi }{12}$ and (c) $\theta =-\frac{\pi }{12}$ . All bands share M̃_FC_ = 0.218 *ea*_0_. The pairs differ in which mode carries a displacement and which carries HT activity: **I**, 1­(a_1_) 2­(a_1_), pure FC with both modes displaced (Δ*q* = 1.72, −0.457; M_HT_ = 0); **II**, 3­(a_1_) 4­(a_1_), an undisplaced participant with a displaced partner (Δ*q* = 0, 0.378; M_HT_ = 0.020, 0.003 *e a*_0_); **III**, 5­(a_1_) 6­(a_1_), a displaced participant with an undisplaced but HT-active spectator (Δ*q* = 0.601, 0; M_HT_ = 0.018, 0.011 *ea*_0_); and **IV**, 5­(a_1_) 7­(a_1_), the same displaced participant with an undisplaced and HT-silent spectator (Δ*q* = 0.601, 0; M_HT_ = 0.018, 0 *ea*_0_). In panel (a), bands I and II are exactly mirror-symmetric, since neither carries FC–HT interference in the separable limit, whereas III and IV already display the asymmetry of eq 20. Mixing breaks the symmetry of I and II and reverses its direction between (b) and (c), the first-order correction being odd in θ; for III, the baseline asymmetry is retained and only modulated. Band IV is unchanged across all three panels because the first-order correction is strictly proportional to the HT prefactor of the partner mode, eq 69, so a rotation acting between two coordinates of no electronic contrast produces no imbalance.

The origin of these different responses can be better understood by examining the leading consequence of such mixing in the smallest nontrivial setting, namely, a two-mode subspace spanned by two totally symmetric normal coordinates.

Let **q** = [*q*_A_, *q*_B_]^T^ denote two ground-state normal coordinates measured from the S_0_ equilibrium geometry, and let $q^{\prime }={[q_{A}^{\prime },q_{B}^{\prime }]}^{T}$ be the corresponding excited-state coordinates measured from the S_1_ equilibrium geometry. With **K** = [Δ*q*_A_, Δ*q*_B_]^T^ the shift vector of eq 3, the two bases are related by

51 $$q^{\prime }=Ĵ\left(q-K\right),,Ĵ=\left[\begin{array}{cc} \cos\theta & \sin\theta \\ -\sin\theta & \cos\theta \end{array}\right]$$

where θ is the Duschinsky mixing angle. At θ = 0, *Ĵ* reduces to the identity, and eq 51 becomes a pure translation, recovering the displaced-oscillator framework.

The exact multidimensional FC–HT integrals can be evaluated by recurrence or generating function methods, but they no longer reduce to the simple one-dimensional parity structure of the AS limit, and the resulting expressions obscure the physical origin of the asymmetry. A perturbative expansion in θ retains analytical transparency while capturing the leading correction.

The complete derivation is given in the Supporting Information; here only the core expressions are quoted.

#### 4.4.1 First-Order Expansion

Alongside the FC and HT overlaps, F_X_ and H_X_ of eqs 7–8 and the ratio γ_*X*_ of eq 16, one further one-mode quantity is required: the logarithmic derivative of F_X_ with respect to the signed displacement, λ_X_ ≡ (∂F_X_/∂Δ*q*_X_)/F_X_. It measures how sharply the FC overlap responds to a small change in geometry along mode *X* and can be expressed as

52 $$\lambda_{X}=\frac{\left|{\Delta \nu }_{X}\right|}{{\Delta q}_{X}}-{\Delta q}_{X}\left(\frac{1}{2}+\frac{L_{m_{X}-1}^{\left(|{\Delta \nu }_{X}|+1\right)}\left(\alpha_{X}\right)}{L_{m_{X}}^{\left(|{\Delta \nu }_{X}|\right)}\left(\alpha_{X}\right)}\right),{,m}_{X}=\min\left(u_{X},w_{X}\right)$$

following the Laguerre convention of Section 2.2, where the single index m_X_ covers either sign of Δν_X_.

The zeroth- and first-order corrections to the asymmetry factor involve the same combinations of one-mode quantities repeatedly. It is therefore convenient to collect these combinations into three composite scalars. Defining the shifted ratio

53 $$\gamma_{X}^{\pm }\equiv \gamma_{X}\pm \frac{{\Delta q}_{X}}{2}$$

the two purely nuclear combinations are

54 $$G^{\pm }=\lambda_{B}\gamma_{A}^{\pm }-\lambda_{A}\gamma_{B}^{\pm }$$

55 $$E^{\pm }=\lambda_{A}\lambda_{B}-\gamma_{A}^{\pm }\gamma_{B}^{\pm }$$

while the corresponding HT contribution is conveniently represented by

56 $$D=\frac{M_{\mathrm{HT},B}}{{\Delta q}_{B}}-\frac{M_{\mathrm{HT},A}}{{\Delta q}_{A}}$$

Every zeroth- and first-order term in the expansion carries the common one-mode product F_A_F_B_, which may therefore be factored out of both branches,

57 $$A\left(\theta \right)=F_{A}F_{B}\left(\alpha_{0}+\theta \alpha_{1}\right),,E\left(\theta \right)=\sigma_{A}\sigma_{B}F_{A}F_{B}\left(\varepsilon_{0}+\theta \varepsilon_{1}\right)$$

with zeroth-order amplitudes

58 $$\alpha_{0}={\mathrm{M̃}}_{\mathrm{FC}}+M_{\mathrm{HT},A}\gamma_{A}+M_{\mathrm{HT},B}\gamma_{B}={\mathrm{M̃}}_{\mathrm{FC}}\left(1+\kappa \right)$$

59 $$\varepsilon_{0}={\mathrm{M̃}}_{\mathrm{FC}}-M_{\mathrm{HT},A}\gamma_{A}-M_{\mathrm{HT},B}\gamma_{B}={\mathrm{M̃}}_{\mathrm{FC}}\left(1-\kappa \right)$$

in which κ is exactly the reduced variable of the combination band (eq 48). The θ = 0 limit therefore reproduces the combination-band amplitudes of eqs 46 and 47 identically, and

60 $$\zeta^{\left(0\right)}=\frac{{\alpha_{0}}^{2}-{\varepsilon_{0}}^{2}}{{\alpha_{0}}^{2}+{\varepsilon_{0}}^{2}}=\frac{2\kappa }{1+\kappa^{2}}$$

recovering eq 20 without further assumption. The first-order corrections reduce to

61 $$\alpha_{1}=\alpha_{0}G^{-}+E^{-}D$$

62 $$\varepsilon_{1}=-\varepsilon_{0}G^{+}+E^{+}D$$

The relative sign between the two lines is not a matter of convention. The HT groups of both branches reduce to $\gamma_{X}G^{\pm }\mp E^{\pm }/{\Delta q}_{X}$ with the same orientation, whereas the FC bracket of the emission branch is $\lambda_{A}\gamma_{B}^{+}-\lambda_{B}\gamma_{A}^{+}=-G^{+}$ . The reversal is therefore the same FC–HT parity relation, now carried to first order in the mixing angle.

The two brackets combine simply in the first-order asymmetry. Writing $G\equiv \frac{1}{2}\left(G^{+}+G^{-}\right)=\lambda_{B}\gamma_{A}-\lambda_{A}\gamma_{B}$ , the first-order asymmetry becomes

63 $$\zeta^{\left(1\right)}=\frac{4\alpha_{0}\varepsilon_{0}}{{\left({\alpha_{0}}^{2}+{\varepsilon_{0}}^{2}\right)}^{2}}\left[2\alpha_{0}\varepsilon_{0}G+D\left(\varepsilon_{0}E^{-}-\alpha_{0}E^{+}\right)\right]$$

Equation 63 separates the two routes by which mode mixing can generate asymmetry. The first term is geometric and is controlled by G , which measures the difference in displacement character between the two normal coordinates. The second term is electronic and is weighted by D , the difference of the HT prefactors per unit displacement, and vanishes whenever M_HT,A_/Δ*q*_A_ = M_HT,B_/Δ*q*_B_, that is, whenever the two coordinates present no HT contrast for the rotation to exploit.

It should be noted that eqs 61 and 62 are written for equal ground- and excited-state frequencies. For ω_A_ ≠ ω_B_ the dimensionless Duschinsky mixing scales as $r=\sqrt{\omega_{A}/\omega_{B}}$ , and the only modification is that each A → B cross term in α_1_ and ε_1_ acquires a factor *r* and each B → A term a factor *r* ^–1^; the zeroth-order amplitudes α_0_, ε_0_ and hence ζ^(0)^ are unchanged.

#### 4.4.2 Regimes of the Duschinsky-Induced Asymmetry

Within the two-mode subspace, each normal mode carries two independent attributes. It is either a *participant*, meaning that its vibrational quantum number changes across the transition, Δν ≠ 0, or a *spectator* with Δν = 0, and, independently, it is either *displaced*, with Δ*q* ≠ 0, or *undisplaced*, with Δ*q* = 0, between the two electronic minima. The four resulting characters, combined across the two modes, generate the physically distinct regimes of the asymmetry.

In each regime, ζ separates into a baseline ζ^(0)^, already present in the displaced-oscillator adiabatic shift limit, and a first-order Duschinsky correction *θ ζ* ^(1)^ that grows linearly with the mixing angle. Three combinations produce a distinctive signature and are treated below, namely the pure FC limit, a participant held at zero displacement, and a displaced participant. The remaining combinations carry no asymmetry, since an all-spectator band is mirror-symmetric to all orders, while two undisplaced participants render the band intensity forbidden, and thus are not shown. Figure 8 compiles ζ­(θ) for the cases below. In every panel, the first-order expansion, shown as the dashed curve, tracks the exact two-mode result, shown as the solid curve, at small θ, with the two separating only once second-order terms in the rotation become appreciable.

**Figure 8:**
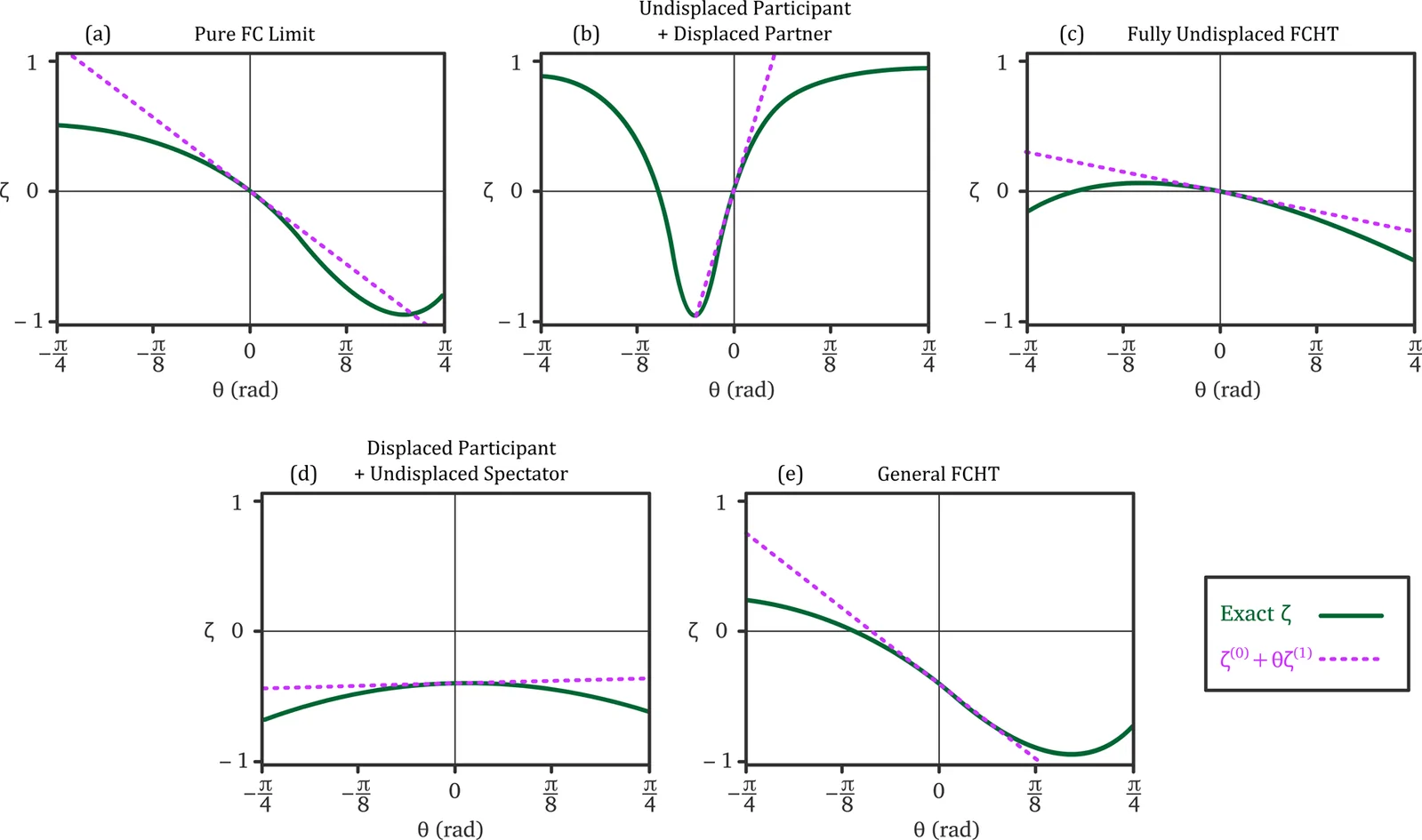
The asymmetry factor ζ as a function of the Duschinsky mixing angle θ, with electronic prefactors and frequencies taken from Mode A = 4­(a_g_) (in-plane C–H bending) and Mode B = 2­(a_g_) (in-plane ring deformation) modes of Anthracene. Throughout, mode A is the participant (0 → 1, Δν_A_ = 1) and mode B is the partner (0 → 0, Δν_B_ = 0), with M_FC_ ∼ −0.982 *ea*_0_, M_HT,A_ ∼ 0.030 *ea*_0_, and M_HT,B_ ∼ −0.064 *ea*_0_; any displaced mode takes Δ*q*_A_ ∼ −0.136 or Δ*q*_B_ ∼ 0.196, and an undisplaced mode takes Δ*q* = 0. The regimes differ only in which modes carry a displacement: (a) pure FC (M_HT,A_ = M_HT,B_ = 0, both displaced); (b) the participant undisplaced, the partner displaced; (c) both modes undisplaced; (d) the participant displaced, the spectator undisplaced; and (e) both modes displaced. Solid green is the exact two-mode result; dashed magenta is the first-order expansion ζ^(0)^ + *θ ζ* ^(1)^.

##### 4.4.2.1 Pure Franck–Condon

When M_HT,A_ = M_HT,B_ = 0, the transition is purely FC. Then κ = 0, D=0 and α_0_ = ε_0_ = M_FC_, so ζ^(0)^ = 0 and eq 63 collapses to the geometric term alone,

64 $$\zeta^{\left(1\right)}=2G=2\left(\lambda_{B}\gamma_{A}-\lambda_{A}\gamma_{B}\right)$$

Mode mixing therefore revives an asymmetry at first order even in the complete absence of HT coupling. Since the electronic channel is closed (D=0) , ζ^(1)^ can only arise through the geometric channel that remains. The result is antisymmetric under A ↔ B, reflecting that the rotation can tip the asymmetry in either direction depending on which mode lends its character to the other. Within this regime, ζ^(1)^ vanishes whenever the two modes present no geometric contrast for the rotation to act on. Either both modes are spectators such that γ_X_ = 0, or a participant is paired with an undisplaced partner, which kills the cross-term. Both conditions reduce to the same point. G measures the difference in vibrational character between the two coordinates, and where that difference is zero, the rotation produces no imbalance between absorption and emission, however strongly it mixes the modes. Displacement and a vibrational quantum change are therefore both required, with the former establishing the geometric contrast and the latter providing the phase asymmetry.

##### 4.4.2.2 An Undisplaced Participant

Let mode A be a participant with Δ*q*_A_ = 0. Since F_A_ → 0, the reduced amplitudes diverge, and the asymmetry must be obtained from the unnormalized amplitudes before the limit is taken. The band retains intensity only through the HT term, which, in the equal-frequency harmonic limit,^30^ is nonzero only for |Δν_A_| = 1 and M_HT,A_ ≠ 0. The zeroth-order amplitudes satisfy α_0_ = −ε_0_, so ζ^(0)^ = 0, and the rotation revives an asymmetry odd in θ,

65 $$\zeta^{\left(1\right)}=-\frac{2}{{\Delta q}_{B}}\frac{M_{\mathrm{HT},B}}{M_{\mathrm{HT},A}}\left({\Delta \nu }_{A}\gamma_{B}+\lambda_{B}\right)\left({\Delta \nu }_{A}+{\Delta \nu }_{B}\right)-{\Delta \nu }_{A}\frac{{\mathrm{M̃}}_{\mathrm{FC}}}{M_{\mathrm{HT},A}}{\Delta q}_{B}$$

Mode A acts as the intensity source, since its HT coupling is the sole reason the band exists; the rotation then leaks amplitude into B’s coordinate, and whether that amplitude generates asymmetry depends on what electronic character B can offer in return. Both terms of eq 65 are electronic in character, with each weighted by a ratio of electronic prefactors, and neither survives if mode B offers no electronic contrast for the rotation to exploit. The first transduces the leaked amplitude through B’s own HT coupling, weighted by M_HT,B_/M_HT,A_, which measures how strongly the transition dipole responds to motion along B relative to A. The second routes it through B’s displacement, weighted by M̃_FC_/M_HT,A_, and it enters with the opposite sign. The two compete, so the asymmetry is sensitive to both the magnitude and the relative sign of M_HT,B_ and M̃_FC_, a further manifestation of the FC–HT interference established for the single-mode case in Section 4.2.1.

Equation 65 carries a “selection rule” for the HT subchannel with no counterpart in the AS limit. ζ^(1)^ collapses entirely to the FC channel when

66 $${\Delta \nu }_{A}+{\Delta \nu }_{B}=0$$

that is, when the combination band conserves the total number of vibrational quanta. One way to interpret this is that the rotation induces an effective transition in B with a spacing of Δν_A_ + Δν_B_, whose vanishing value closes the HT subchannel regardless of how strongly B is electronically active.

When the partner is instead an undisplaced spectator, γ_B_ = 0 and the FC pathway is closed, reducing eq 65 to

67 $$\zeta^{\left(1\right)}={\Delta \nu }_{A}\frac{M_{\mathrm{HT},B}}{M_{\mathrm{HT},A}}\left(2u_{B}+1\right)$$

Only the ratio of the two HT prefactors survives. If mode B is HT-inactive, the two coordinates become electronically indistinguishable under rotation and ζ^(1)^ vanishes, since mode mixing alone produces no imbalance.

##### 4.4.2.3 A Displaced Participant

When a participant (mode A) is displaced, Δ*q*_A_ ≠ 0, it carries FC intensity in its own right, and the single-mode FC–HT interference produces a static asymmetry ζ^(0)^ ≠ 0 already at the AS level. If the partner is also displaced, be it a spectator or a participant, no special structure survives and one simply recovers the general result, eq 63.

In the special case where the partner is an undisplaced spectator, Δ*q*_B_ = 0 and Δν_B_ = 0, the baseline reduces to the single-mode amplitudes of the displaced participant,

68 $$\alpha_{0}={\mathrm{M̃}}_{\mathrm{FC}}+M_{\mathrm{HT,A}}\gamma_{A},{,\varepsilon }_{0}={\mathrm{M̃}}_{\mathrm{FC}}-M_{\mathrm{HT,A}}\gamma_{A}$$

Since γ_B_ = 0 and λ_B_ = 0, the geometric bracket vanishes, G→0 , and the whole correction is carried by the electronic channel:

69 $$\zeta^{\left(1\right)}=\frac{2M_{\mathrm{HT},B}\alpha_{0}\varepsilon_{0}\left\{\alpha_{0}\left[\gamma_{A}^{+}+\left(2u_{B}+1\right)\lambda_{A}\right]+\varepsilon_{0}\left[\gamma_{A}^{-}-\left(2u_{B}+1\right)\lambda_{A}\right]\right\}}{{\left({\alpha_{0}}^{2}+{\varepsilon_{0}}^{2}\right)}^{2}}$$

The correction is directly proportional to M_HT,B_. If the spectator is HT-inactive, the rotation lacks electronic contrast, making the asymmetry invariant under mode mixing. Even when the spectator is HT-active, the prefactor α_0_ε_0_ acts as a further suppressor, since, as ζ^(0)^ saturates toward ± 1, one member of the cross-swap pair is extinguished, α_0_ε_0_ → 0, and the rotation has progressively less room to modulate the asymmetry.

Taken together, the three regimes illustrate a general principle. Duschinsky rotation sets the geometric coupling between modes, but whether that coupling generates an imbalance depends on which of the two channels in eq 63 it can open. The electronic channel, weighted by D , requires a contrast in HT electronic prefactors between the modes and closes when the two coordinates are electronically indistinguishable under the rotation. The geometric channel, weighted by G , requires no HT activity at all and closes only when the rotation has no geometric contrast to exploit, that is, unless at least one mode is a participant and its partner is displaced. Where either channel is open, the rotation converts the corresponding contrast into a measurable imbalance between absorption and emission, gated in both cases by the common factor α_0_ε_0_, which suppresses the correction as ζ^(0)^ saturates toward ±1.

### 4.5 Many-Mode Limit and Overall Physical Picture

The two-mode treatment captures the essential way in which Duschinsky rotation modifies FC–HT absorption–emission asymmetry. Its extension to a larger set of totally symmetric modes is straightforward in the small-rotation limit. After fixing the arbitrary signs of the normal coordinates, the Duschinsky matrix may be written as an orthogonal rotation in the active normal-coordinate subspace,

70 Ĵ=exp(Φ̂)

where Φ̂ is antisymmetric and can be decomposed into elementary planar generators,

71 $$\mathrm{\Phi ̂}=\underset{X<Y}{\sum }\theta_{\mathrm{XY}}{\mathrm{R̂}}_{\mathrm{XY}},,({\mathrm{R̂}}_{\mathrm{XY}}{)}_{ij}=\delta_{iX}\delta_{jY}-\delta_{iY}\delta_{jX}$$

Here θ_XY_ is the mixing angle in the plane spanned by modes *X* and *Y*. Expanding the exponential gives

72 $$Ĵ=Î+\underset{X<Y}{\sum }\theta_{\mathrm{XY}}{\mathrm{R̂}}_{\mathrm{XY}}+O\left(\theta^{2}\right)$$

Thus, at leading order, the noncommutativity of rotations in different mode planes is irrelevant. Couplings between distinct planar rotations arise only through products of generators, and therefore only enter at second order and higher.

This is why the two-mode result is not merely a special case, but the elementary building block of the general problem. In the AS limit, the multidimensional FC and HT integrals factorize into one-mode contributions. Differentiating with respect to a single angle θ_XY_ perturbs only the pair of modes X and Y, while all other modes remain as spectator factors. The many-mode asymmetry can therefore be approximated as

73 $$\zeta \approx \zeta^{\left(0\right)}+\underset{X<Y}{\sum }\theta_{\mathrm{XY}}\zeta_{\mathrm{XY}}^{\left(1\right)}$$

where ζ^(0)^ is the FC–HT asymmetry already present in the AS limit, and ζ_XY_^(1)^ is the first-order Duschinsky correction associated with mixing the particular pair of modes X and Y. Each ζ_XY_^(1)^ is therefore described by the same two-mode physics developed above. It is worth noting that different pairwise corrections can have different signs. Duschinsky rotation does not simply increase the asymmetry as more modes are included; different mode pairs may reinforce or cancel one another.

The central conclusion, therefore, is that the apparently many-dimensional Duschinsky problem remains approximately reducible to the simple two-mode mixing limit at first order. Duschinsky rotation, in the context of spectral asymmetry, can be interpreted as redistributing FC and HT character between pairs of normal coordinates and modifying the same interference pattern already present in the displaced-oscillator picture, rather than acting through an independent channel. Genuine irreducible many-mode effects enter only at significant degrees of mode mixing.

## 5 Conclusion

This work develops a unified account of Franck–Condon–Herzberg–Teller (FC–HT) interference as a source of absorption–emission asymmetry, from the displaced harmonic-oscillator limit through to leading-order Duschinsky rotation. The organizing result is that, in the separable limit at equal ground- and excited-state frequencies, the FC and HT vibrational integrals are not merely related by parity, but are strictly proportional, with a constant fixed by the vibrational spacing and the signed displacement alone. The asymmetry factor consequently collapses onto a single, one-parameter curve in the reduced variable (κ), which separates cleanly into an electronic ratio of the HT and shifted FC prefactors and a nuclear ratio of spacing to displacement. The central parity result of Kundu and Roy is recovered as the natural baseline of this description^8,19^ and the remaining features of the asymmetry follow from where κ vanishes, diverges, or passes through unity.

Several consequences follow from resolving the interference in terms of the signed normal-coordinate displacement rather than only through the Huang–Rhys factor. For HT-active, totally symmetric modes, reversing the displacement direction reverses the interference direction, except where the midpoint-shifted FC prefactor itself passes through zero, which, within the fixed-reference expansion, is the only electronic sign change available to a totally symmetric mode. For nondegenerate, nontotally symmetric modes, FC–HT interference is suppressed at the symmetry-preserving geometry, but can reappear under symmetry-breaking displacement. Within the fixed-reference linear-HT model, its direction is then determined solely by the vibrational quantum change. The analysis also shows that, although absolute transition strengths depend on the specific vibronic levels involved, the normalized asymmetry depends only on the vibrational spacing within the displaced-oscillator limit. Extension to combination bands further shows that multiple HT pathways can interfere with one another, producing internal nodal boundaries and sign inversions that have no single-mode analogue.

The same reduction points to how the framework might be applied to measured spectra. Although a displacement scan is a theoretical construction, a vibronic progression traverses the same universal curve in the one variable an experiment can vary, the vibrational spacing, at fixed geometry and hence at fixed electronic prefactors. This lifts a degeneracy that no isolated line can, allowing us to distinguish between weak coupling at larger displacement and strong coupling at smaller displacement, such that the ratio of electronic prefactors and displacement can be estimated from asymmetry alone. We demonstrate this strategy using the jet-cooled S_0_ ↔ S_1_ spectrum of phenanthrene.^31^ For mode 22­(a_1_), the measured progression resolves the reciprocal-τ ambiguity. More importantly, when the same progression-based analysis is applied across the low-frequency totally symmetric modes, the relative HT coupling strengths recovered from the asymmetry agree remarkably closely with those obtained from the explicit first-order spectral parametrization of Warren et al.,^31^ differing by only 0.9% and 5.0% relative to mode 20­(a_1_) for mode 21­(a_1_) and 22­(a_1_) respectively. Thus, a substantially reduced analysis based on the asymmetry pattern and equilibrium displacements recovers essentially the same mode-to-mode HT information as a full first-order fit, demonstrating that the universal variable is not only an organizing principle for the theory, but also a practical route for extracting electronic coupling information from experimental spectra.

The perturbative Duschinsky treatment clarifies how these idealized rules are modified by mode mixing, and does so within the same description rather than as a separate problem. Expanding in the mixing angle leaves the zeroth-order amplitudes exactly those of the unrotated limit, so that κ survives intact and the rotation enters as a correction to a baseline already characterized. Importantly, the correction itself closes on the same one-mode quantities as those used in the AS framework. The asymmetry from mode mixing can be interpreted as arising from two separate pathways: an electronic one, weighted by the contrast in HT prefactors per unit displacement between the mixed coordinates, and a geometric one, weighted by the difference in their vibrational character. Both are gated by a common factor that vanishes as one member of the cross-swap pair is extinguished, so the rotation has progressively less room to act as the baseline asymmetry saturates. Mode mixing therefore redistributes FC and HT character between coordinates rather than introducing any new electronic quantity, and generates asymmetry only where one of these contrasts already exists. In the pure FC limit, the electronic channel closes and the geometric one survives alone, yielding an analogue of FC–HT interference in which asymmetry arises between mixed FC overlap pathways even in the complete absence of HT coupling. In the small-rotation limit, the many-mode problem reduces to a sum of pairwise corrections of this form, which may reinforce or cancel one another.

These results should be viewed as symmetry and perturbative statements rather than as direct quantitative rules for congested molecular spectra. The proportionality on which the reduction rests is specific to the equal-frequency separable limit since frequency changes introduce a squeezing contribution that reinstates a dependence on the absolute vibronic level. Anharmonicity, which plays no role in this analysis, can contribute to mirror-symmetry breaking on a scale comparable to or larger than the effects treated here. Overlapping vibronic progressions can further obscure the patterns. While the asymmetry factor of an individual vibronic transition is influenced by many competing parameters and is therefore difficult to interpret in isolation, the spectrum as a whole carries a collective asymmetry signature. Within this framework, that signature can be used to disentangle contributions step by step, allowing electronic, geometric, and mode-mixing effects to be identified progressively rather than inferred from any single transition.

## Supplementary Material





## Notes

Analytical derivation of the leading asymmetry induced by Duschinsky rotation (PDF)
Supporting computational data for reproducibility including molecular orientations, mass-weighted Cartesian displacement vectors, additional displacement scans (ZIP)
