# Supplementary material for: A Theoretical Study of Absorption–Emission Asymmetry from Franck–Condon–Herzberg–Teller Interference and Duschinsky Mixing
Source: J Phys Chem A. 2026 Oct 13;130(42):8249–67. doi: 10.1021/acs.jpca.6c04260 (PMC13700109; doi:10.1021/acs.jpca.6c04260)
Supplement: Supplementary file 1 [file jp6c04260_si_001.pdf]

A Theoretical Study of Absorption–Emission Asymmetry from Franck–Condon–Herzberg–Teller Interference and Duschinsky Mixing (Supporting Information)

Cedric Poon[1] and Chun Hey Siu[2]

[1]Department of Chemistry, Imperial College London, London SW7 2AZ, United Kingdom
[2]Department of Chemistry, The Hong Kong University of Science and Technology, Clear Water Bay, Kowloon, Hong Kong

1 Notation and the one-mode blocks

1.1 The midpoint coordinate under mode mixing

In the AS limit the midpoint coordinate of Eq. (M-2) is the mean of the two equilibrium values of a single coordinate, $q_{\mathrm{M}} = q - \frac{1}{2}(q_{\mathrm{eq,GS}} + q_{\mathrm{eq,ES}})$. Once Duschinsky rotation is admitted, mode X of one state is a linear combination of several modes of the other and $q_{\mathrm{eq,ES}}$ has no meaning as a component. The construction survives because a midpoint is an affine notion and only the positions of the two minima are required, not the axes through them. Placing the S_0 minimum at $\mathbf{q} = \mathbf{0}$ and the S_1 minimum at $\mathbf{q} = \mathbf{K}$ in the S_0 normal-coordinate frame, with $\mathbf{K} = [\Delta q_{\mathrm{A}}, \Delta q_{\mathrm{B}}]^{\top}$ the shift vector of Eq. (M-3),

$$q_{\mathrm{M}} \equiv \mathbf{q} - \frac{\mathbf{K}}{2}, \tag{1}$$

which is componentwise identical to Eq. (M-2) but refers only to $\mathbf{K}$, and is therefore defined at every mixing angle.

Equation (1) is the convention in which the electronic prefactors are already expressed. The HT prefactors are derivatives of the transition dipole along the S_0 normal coordinates, so that, as in Eqs. (M-42)–(M-43),

$$\mathrm{M}(\mathbf{q}) = \mathrm{M}_{\mathrm{FC}} + \sum_{X} \mathrm{M}_{\mathrm{HT,X}}\,(q_X - \Delta q_{\mathrm{X}}) = \widetilde{\mathrm{M}}_{\mathrm{FC}} + \sum_{X} \mathrm{M}_{\mathrm{HT,X}}\, q_{\mathrm{M,X}}. \tag{2}$$

Two properties of this frame are used repeatedly below. First, $\mathbf{K}$ is fixed by the positions of the two minima and does not depend on θ, so the ground-state factors and the weight $q_{\mathrm{M,X}}$ are θ-independent and the entire θ dependence resides in the excited-state factors. Second, Eq. (1) symmetrises the displacement but not the mixing and no bisecting frame $\hat{J}^{1/2}$ is used. The second property is why the absorption and emission expansions of Section 3 are not related by a single change of sign.

1.2 The one-mode blocks

The blocks defined here are one-dimensional, θ-independent objects evaluated in the AS geometry, where the two oscillators of a given mode share an axis and the arguments are unambiguous:

$$q_{\mathrm{GS,X}} \equiv q_{\mathrm{M,X}} + \frac{\Delta q_{\mathrm{X}}}{2}, \qquad q_{\mathrm{ES,X}} \equiv q_{\mathrm{M,X}} - \frac{\Delta q_{\mathrm{X}}}{2}. \tag{3}$$

For a weight operator $\hat{\Omega}(q_{\mathrm{M}})$,

$$\langle \hat{\Omega} \rangle_X \equiv \int \chi_{\mathrm{GS}:u_X}(q_{\mathrm{GS,X}})\, \hat{\Omega}(q_{\mathrm{M,X}})\, \chi_{\mathrm{ES}:w_X}(q_{\mathrm{ES,X}})\, \mathrm{d}q_{\mathrm{M,X}}. \tag{4}$$

The FC and HT overlaps of Eqs. (M-7)–(M-8) are the cases $\hat{\Omega} = 1$ and $\hat{\Omega} = q_{\mathrm{M}}$, written $\mathrm{F_X}$ and $\mathrm{H_X}$. Three further quantities are required, the second-moment overlap and the two displacement derivatives,

$$\mathrm{Z_X} \equiv \langle q_{\mathrm{M,X}}^2 \rangle_X, \qquad \Lambda_{\mathrm{X}} \equiv \frac{\partial \mathrm{F_X}}{\partial \Delta q_{\mathrm{X}}}, \qquad \Xi_{\mathrm{X}} \equiv \frac{\partial \mathrm{H_X}}{\partial \Delta q_{\mathrm{X}}}. \tag{5}$$

Since $\mathrm{F_X}$ and $\mathrm{H_X}$ depend on the geometry only through Δq_{X}, these derivatives are unambiguous.

Two derivative-type integrals arise in the expansion, in which $\partial/\partial q_{\mathrm{M}}$ acts on the excited-state factor. Differentiating Eq. (4) and integrating by parts, the harmonic-oscillator boundary terms vanishing at infinity, gives

$$\int \chi_{\mathrm{GS}:u_X}(q_{\mathrm{GS,X}})\, \chi'_{\mathrm{ES}:w_X}(q_{\mathrm{ES,X}})\, \mathrm{d}q_{\mathrm{M,X}} = -\Lambda_{\mathrm{X}},$$

$$\int \chi_{\mathrm{GS}:u_X}(q_{\mathrm{GS,X}})\, q_{\mathrm{M,X}}\, \chi'_{\mathrm{ES}:w_X}(q_{\mathrm{ES,X}})\, \mathrm{d}q_{\mathrm{M,X}} = -\left(\Xi_{\mathrm{X}} + \tfrac{1}{2}\mathrm{F_X}\right). \tag{6}$$

2 First-order expansion of the absorption integrals

The two-mode absorption integral with weight $\hat{\Omega}$ is

$$I^{(\mathrm{abs})}_{\hat{\Omega}}(\theta) = \iint \chi_{\mathrm{GS}:u_A}(q_{\mathrm{GS,A}})\,\chi_{\mathrm{GS}:u_B}(q_{\mathrm{GS,B}})\,\hat{\Omega}\,\chi_{\mathrm{ES}:w_A}(q'_A)\,\chi_{\mathrm{ES}:w_B}(q'_B)\,\mathrm{d}q_{\mathrm{M,A}}\,\mathrm{d}q_{\mathrm{M,B}}, \tag{7}$$

Eq. (M-49) reads $\mathbf{q}' = \hat{J}(q_{\mathrm{M}} - \mathbf{K}/2)$. Hence

$$v_X \equiv q_{\mathrm{M,X}} - \frac{\Delta q_{\mathrm{X}}}{2} \tag{8}$$

is the value taken by q'_X at $\theta = 0$. Differentiating Eq. (M-49) at $\theta = 0$,

$$\left.\frac{\partial q'_A}{\partial \theta}\right|_0 = v_B, \qquad \left.\frac{\partial q'_B}{\partial \theta}\right|_0 = -v_A, \tag{9}$$

so that the chain rule gives

$$\left.\frac{\partial}{\partial \theta}\left[\chi_{\mathrm{ES}:w_A}(q'_A)\,\chi_{\mathrm{ES}:w_B}(q'_B)\right]\right|_0 = v_B\,\chi'_{\mathrm{ES}:w_A}(v_A)\,\chi_{\mathrm{ES}:w_B}(v_B) - v_A\,\chi_{\mathrm{ES}:w_A}(v_A)\,\chi'_{\mathrm{ES}:w_B}(v_B). \tag{10}$$

The rotation therefore differentiates one excited-state wavefunction and weights the other coordinate by v. Substituting Eq. (10) into Eq. (7), using

$$\langle v\rangle_X = \mathrm{H_X} - \frac{\Delta q_{\mathrm{X}}}{2}\mathrm{F_X}, \qquad \langle q_{\mathrm{M}} v\rangle_X = \mathrm{Z_X} - \frac{\Delta q_{\mathrm{X}}}{2}\mathrm{H_X}, \tag{11}$$

and eliminating the derivative integrals with Eq. (6), gives

$$\mathrm{F^{(abs)}} \simeq \mathrm{F_A F_B} + \theta\left[\Lambda_{\mathrm{B}}\left(\mathrm{H_A} - \frac{\Delta q_{\mathrm{A}}}{2}\mathrm{F_A}\right) - \Lambda_{\mathrm{A}}\left(\mathrm{H_B} - \frac{\Delta q_{\mathrm{B}}}{2}\mathrm{F_B}\right)\right], \tag{12}$$

$$\mathrm{H}^{(\mathrm{abs})}_A \simeq \mathrm{H_A F_B} + \theta\left[\Lambda_{\mathrm{B}}\left(\mathrm{Z_A} - \frac{\Delta q_{\mathrm{A}}}{2}\mathrm{H_A}\right) - \left(\Xi_{\mathrm{A}} + \frac{\mathrm{F_A}}{2}\right)\left(\mathrm{H_B} - \frac{\Delta q_{\mathrm{B}}}{2}\mathrm{F_B}\right)\right], \tag{13}$$

$$\mathrm{H}^{(\mathrm{abs})}_B \simeq \mathrm{H_B F_A} + \theta\left[\left(\Xi_{\mathrm{B}} + \frac{\mathrm{F_B}}{2}\right)\left(\mathrm{H_A} - \frac{\Delta q_{\mathrm{A}}}{2}\mathrm{F_A}\right) - \Lambda_{\mathrm{A}}\left(\mathrm{Z_B} - \frac{\Delta q_{\mathrm{B}}}{2}\mathrm{H_B}\right)\right]. \tag{14}$$

Each first-order term factorises into a derivative block on one mode and a v-weighted block on the other. The second-moment overlap $\mathrm{Z_X}$ enters only where the rotation acts on a mode that already carries the HT weight, so that two factors of the midpoint coordinate meet on the same mode.

3 Emission by label exchange

For emission the electronic labels are exchanged, and no separate expansion is required. The emission integrand is the same function of q_{M} as the absorption integrand with the vibrational labels interchanged, and the midpoint weight is fixed by the geometry and unaffected by which factor carries the initial quanta. Hence, exactly and at every θ,

$$\mathrm{F^{(emi)}}(\theta; u, w) = \mathrm{F^{(abs)}}(\theta; w, u), \qquad \mathrm{H}^{(\mathrm{emi})}_X(\theta; u, w) = \mathrm{H}^{(\mathrm{abs})}_X(\theta; w, u). \tag{15}$$

Equation (15) is a statement about labels rather than geometry, so it invokes no reflection and no excited-state axis.

It therefore suffices to know how the one-mode blocks respond to $u_X \leftrightarrow w_X$. These are the θ-independent objects of Eq. (4), evaluated in the AS geometry, and the reflection argument is applied only here. Under the exchange the two oscillators swap position, corresponding to the reflection $q_{\mathrm{M}} \to -q_{\mathrm{M}}$ and nothing more. Using $\chi_n(-y) = (-1)^n \chi_n(y)$, the two wavefunctions together supply $(-1)^{(\mathrm{u_X + w_X})}$ while the weight supplies its own parity. Hence for a weight of definite parity, $\hat{\Omega}(-q_{\mathrm{M}}) = \pm\hat{\Omega}(q_{\mathrm{M}})$

$$\overline{\langle\hat{\Omega}\rangle}_X = \pm\,\sigma_X\,\langle\hat{\Omega}\rangle_X, \qquad \sigma_X = (-1)^{\Delta v_X} = (-1)^{u_X + w_X}, \tag{16}$$

the sign being that of the weight. The three weights required are $\hat{\Omega} = 1, q_{\mathrm{M}}, q_{\mathrm{M}}^2$, of parity $+$, $-$ and $+$ respectively. The first two cases are the opposite-parity relation, which is what started the FCHT interference. Since Λ_{X} and Ξ_{X} are derivatives with respect to Δq_{X}, and Eq. (16) holds at every Δq_{X} while σ_{X} does not depend on it, they inherit the parities of $\mathrm{F_X}$ and $\mathrm{H_X}$. Hence

$$\bar{\mathrm{F}}_{\mathrm{X}} = \sigma_{\mathrm{X}}\mathrm{F_X}, \quad \bar{\mathrm{H}}_{\mathrm{X}} = -\sigma_{\mathrm{X}}\mathrm{H_X}, \quad \bar{\mathrm{Z}}_{\mathrm{X}} = \sigma_{\mathrm{X}}\mathrm{Z_X}, \quad \bar{\Lambda}_{\mathrm{X}} = \sigma_{\mathrm{X}}\Lambda_{\mathrm{X}}, \quad \bar{\Xi}_{\mathrm{X}} = -\sigma_{\mathrm{X}}\Xi_{\mathrm{X}}. \tag{17}$$

Substituting Eq. (17) into Eqs. (12)–(14),

$$\mathrm{F}^{(\mathrm{emi})} \simeq \sigma_\mathrm{A}\sigma_\mathrm{B}\left\{\mathrm{F_A F_B} + \theta\left[\Lambda_\mathrm{A}\left(\mathrm{H_B} + \frac{\Delta q_\mathrm{B}}{2}\mathrm{F_B}\right) - \Lambda_\mathrm{B}\left(\mathrm{H_A} + \frac{\Delta q_\mathrm{A}}{2}\mathrm{F_A}\right)\right]\right\}, \tag{18}$$

$$\mathrm{H}_A^{(\mathrm{emi})} \simeq \sigma_\mathrm{A}\sigma_\mathrm{B}\left\{-\mathrm{H_A F_B} + \theta\left[\Lambda_\mathrm{B}\left(\mathrm{Z_A} + \frac{\Delta q_\mathrm{A}}{2}\mathrm{H_A}\right) - \left(\Xi_\mathrm{A} - \frac{\mathrm{F_A}}{2}\right)\left(\mathrm{H_B} + \frac{\Delta q_\mathrm{B}}{2}\mathrm{F_B}\right)\right]\right\}, \tag{19}$$

$$\mathrm{H}_B^{(\mathrm{emi})} \simeq \sigma_\mathrm{A}\sigma_\mathrm{B}\left\{-\mathrm{H_B F_A} + \theta\left[\left(\Xi_\mathrm{B} - \frac{\mathrm{F_B}}{2}\right)\left(\mathrm{H_A} + \frac{\Delta q_\mathrm{A}}{2}\mathrm{F_A}\right) - \Lambda_\mathrm{A}\left(\mathrm{Z_B} + \frac{\Delta q_\mathrm{B}}{2}\mathrm{H_B}\right)\right]\right\}. \tag{20}$$

Relative to absorption, emission carries the overall factor $\sigma_\mathrm{A}\sigma_\mathrm{B}$, the sign reversal of the zeroth-order HT blocks, the replacements $\Delta q_\mathrm{X} \to -\Delta q_\mathrm{X}$ and $\frac{1}{2} \to -\frac{1}{2}$ inside the first-order brackets (and in the FC line only, a global sign change of the first-order term).

4 Reduction of the one-mode quantities

Equations (12)–(20) contain five blocks per coordinate. They are not independent. Introducing the mode-local ratios

$$\gamma_\mathrm{X} = \frac{\mathrm{H_X}}{\mathrm{F_X}}, \qquad \lambda_\mathrm{X} = \frac{\Lambda_\mathrm{X}}{\mathrm{F_X}}, \qquad \xi_\mathrm{X} = \frac{\Xi_\mathrm{X}}{\mathrm{F_X}}, \qquad z_\mathrm{X} = \frac{\mathrm{Z_X}}{\mathrm{F_X}}, \tag{21}$$

the proportionality of Eq. (M-16) applies to each mode separately, so that $\gamma_\mathrm{X} = -\Delta\nu_\mathrm{X}/\Delta q_\mathrm{X}$ exactly as in the AS limit. The last three ratios are defined only where $\mathrm{F_X} \neq 0$; the zeros of $\mathrm{F_X}$ are the zeros of $L_{m_X}^{(|\Delta\nu_\mathrm{X}|)}(\alpha_X)$, at which λ_X, ξ_X and z_X diverge together while the unnormalised blocks remain finite. The ratio γ_X is exempt since $\mathrm{H_X} = \gamma_\mathrm{X}\mathrm{F_X}$ vanishes with $\mathrm{F_X}$, so γ_X retains the finite value $-\Delta\nu_\mathrm{X}/\Delta q_\mathrm{X}$ throughout.

4.1 Three identities

4.1.0.1 Identity I: Differentiating $\mathrm{H_X} = \gamma_\mathrm{X}\mathrm{F_X}$ with respect to Δq_X and using $\partial\gamma_\mathrm{X}/\partial\Delta q_\mathrm{X} = \Delta\nu_\mathrm{X}/\Delta q_\mathrm{X}^2 = -\gamma_\mathrm{X}/\Delta q_\mathrm{X}$,

$$\boxed{\xi_\mathrm{X} = \gamma_\mathrm{X}\left(\lambda_\mathrm{X} - \frac{1}{\Delta q_\mathrm{X}}\right)} \tag{I}$$

4.1.0.2 Identity II: Applying the ladder operator to the weight on the ground-state side,

$$q_\mathrm{M}\,\chi_{\mathrm{GS:u}}(q_\mathrm{GS}) = \tfrac{1}{\sqrt{2}}\left[\sqrt{u}\,\chi_{\mathrm{GS:}u-1}(q_\mathrm{GS}) + \sqrt{u+1}\,\chi_{\mathrm{GS:}u+1}(q_\mathrm{GS})\right] - \tfrac{\Delta q}{2}\,\chi_{\mathrm{GS:u}}(q_\mathrm{GS}), \tag{22}$$

and writing

$$P = \tfrac{1}{\sqrt{2}}\sqrt{u}\,\mathrm{F}^{(u-1;\mathrm{w})}, \qquad Q = \tfrac{1}{\sqrt{2}}\sqrt{u+1}\,\mathrm{F}^{(u+1;\mathrm{w})}, \tag{23}$$

the case $\hat{\Omega} = 1$ reproduces Eq. (M-12) and, with Eq. (M-16), fixes the symmetric combination $P + Q = (\gamma + \frac{\Delta q}{2})\mathrm{F}$. The case $\hat{\Omega} = q_\mathrm{M}$ gives

$$\mathrm{Z} = \tfrac{1}{\sqrt{2}}\left[\sqrt{u}\,\mathrm{H}^{(u-1;\mathrm{w})} + \sqrt{u+1}\,\mathrm{H}^{(u+1;\mathrm{w})}\right] - \tfrac{\Delta q}{2}\mathrm{H}, \tag{24}$$

and applying Eq. (M-16) to each neighbour, whose spacings are $\Delta\nu \pm 1$, leaves $\mathrm{Z} = \gamma^2\mathrm{F} - (P - Q)/\Delta q$. The antisymmetric combination is fixed by Eq. (6): integrating by parts gives $\Lambda = \int \chi'_{\mathrm{GS:u}}(q_\mathrm{GS})\,\chi_{\mathrm{ES:w}}(q_\mathrm{ES})\,\mathrm{d}q_\mathrm{M}$, and $\chi'_{\mathrm{GS:u}} = \frac{1}{\sqrt{2}}[\sqrt{u}\,\chi_{\mathrm{GS:}u-1} - \sqrt{u+1}\,\chi_{\mathrm{GS:}u+1}]$ gives $P - Q = \Lambda$. Hence

$$\boxed{z_\mathrm{X} = \gamma_\mathrm{X}^2 - \frac{\lambda_\mathrm{X}}{\Delta q_\mathrm{X}}}. \tag{II}$$

No recurrence relation is required: the symmetric ladder combination yields H and the antisymmetric one yields Λ, and together they close the system.

4.1.0.3 Identity III: The remaining quantity follows in closed form from the FC overlap of Eq. (M-11). Differentiating logarithmically, the Gaussian contributes $-\Delta q/2$, the prefactor $(\Delta q)^{\Delta\nu}$ contributes $\Delta\nu/\Delta q$, and the chain rule on $\alpha = \Delta q^2/2$ contributes $\Delta q\,L'/L$; with $\mathrm{d}L_n^{(a)}(\alpha)/\mathrm{d}\alpha = -L_{n-1}^{(a+1)}(\alpha)$,

$$\boxed{\lambda_\mathrm{X} = -\gamma_\mathrm{X} - \frac{\Delta q_\mathrm{X}}{2} - \Delta q_\mathrm{X}\,\mathrm{R_X}, \qquad \mathrm{R_X} = \frac{L_{u_X-1}^{(\Delta\nu_\mathrm{X}+1)}(\alpha_X)}{L_{u_X}^{(\Delta\nu_\mathrm{X})}(\alpha_X)}, \qquad \alpha_X = \frac{\Delta q_\mathrm{X}^2}{2}} \tag{III}$$

with $L_{-1}^{(k)} \equiv 0$. Equation (III) is written for $\Delta\nu_\mathrm{X} \geq 0$, as is Eq. (M-11). For $\Delta\nu_\mathrm{X} < 0$ one uses $\lambda^{(u;w)} = \lambda^{(w;u)}$, which follows from Eq. (M-9) because σ_X is independent of Δq_X and cancels in the ratio.

4.2 Three consequences

4.2.0.1 (i) Only γ and λ are fundamental. Z_X and Ξ_X carry no information beyond the FC overlap and its first displacement derivative. Equivalently, γ_X and λ_X are the symmetric and antisymmetric combinations of the same pair of neighbouring FC overlaps,

$$\sqrt{\tfrac{u}{2}}\,\frac{F^{(u-1;w)}}{F} = \tfrac{1}{2}\Big(\gamma + \tfrac{\Delta q}{2} + \lambda\Big), \qquad \sqrt{\tfrac{u+1}{2}}\,\frac{F^{(u+1;w)}}{F} = \tfrac{1}{2}\Big(\gamma + \tfrac{\Delta q}{2} - \lambda\Big), \tag{25}$$

of which the sum is Eq. (M-12). This is also why the reduction of Eq. (M-16) does not extend to λ_X: the proportionality is a property of the symmetric ladder combination that defines H, not a generic overlap identity.

4.2.0.2 (ii) A spectator is inert in Ξ. If $\Delta\nu_X = 0$ then $\gamma_X = 0$ and Eq. (M-16) forces $H_X \equiv 0$ at every displacement; hence $\Xi_X \equiv 0$ and $\xi_X = 0$, displaced or not. If the spectator is in addition undisplaced, direct evaluation of Eq. (4) at $\Delta q_X = 0$ gives the exact values

$$F_X = 1, \qquad H_X = \Lambda_X = \Xi_X = 0, \qquad Z_X = u_X + \tfrac{1}{2}, \tag{26}$$

so that $\gamma_X = \lambda_X = \xi_X = 0$ and $z_X = u_X + \frac{1}{2}$. These are exact statements at $\Delta q_X = 0$, not small-displacement limits.

4.2.0.3 (iii) For $u_X = 0$ everything is explicit. Since R_X vanishes identically for $u_X = 0$,

$$\lambda_X = \frac{\Delta\nu_X}{\Delta q_X} - \frac{\Delta q_X}{2} = -\gamma_X^{+}, \qquad \gamma_X^{\pm} = \gamma_X \pm \frac{\Delta q_X}{2}, \tag{27}$$

noting that $u_X = 0$ forces $\Delta\nu_X \geq 0$. For $w_X = 0$, and hence $\Delta\nu_X \leq 0$, the same argument gives $\lambda_X = +\gamma_X^{-}$. For transitions out of the vibrational ground state, every one-mode quantity in Eqs. (12)–(20) is therefore an explicit function of $\Delta\nu_X$ and Δq_X alone.

5 Reduced amplitudes and their angular slopes

Every zeroth- and first-order term in Eqs. (12)–(20) shares the common product $F_A F_B$, so the transition dipole strengths of the two members of the cross-swap pair may be written as in Eq. (M-55),

$$A(\theta) = F_A F_B\big(\alpha_0 + \theta\alpha_1\big), \qquad E(\theta) = \sigma_A\sigma_B\, F_A F_B\big(\varepsilon_0 + \theta\varepsilon_1\big). \tag{28}$$

Throughout this section the nuclear brackets of Eqs. (M-51)–(M-54) are used,

$$\gamma_X^{\pm} = \gamma_X \pm \frac{\Delta q_X}{2}, \quad \mathbb{G}^{\pm} = \lambda_B\gamma_A^{\pm} - \lambda_A\gamma_B^{\pm}, \quad \mathbb{E}^{\pm} = \lambda_A\lambda_B - \gamma_A^{\pm}\gamma_B^{\pm}, \quad \mathfrak{D} = \frac{M_{HT,B}}{\Delta q_B} - \frac{M_{HT,A}}{\Delta q_A}. \tag{29}$$

5.1 Zeroth order

Collecting the zeroth-order terms of Eqs. (12)–(20) and using $\gamma_X = H_X/F_X$ gives Eqs. (M-56)–(M-57) directly, the sign reversal of the HT blocks in Eqs. (19)–(20) being what distinguishes ε_0 from α_0. Writing them as $\widetilde{M}_{FC}(1 \pm \kappa)$ makes the $\theta = 0$ limit identical to the combination-band result of Eqs. (M-44)–(M-45), and

$$\zeta^{(0)} = \frac{\alpha_0^2 - \varepsilon_0^2}{\alpha_0^2 + \varepsilon_0^2} = \frac{(1+\kappa)^2 - (1-\kappa)^2}{(1+\kappa)^2 + (1-\kappa)^2} = \frac{2\kappa}{1+\kappa^2}, \tag{30}$$

which is Eq. (M-58), recovering the universal curve of Eq. (M-20) with no further assumption.

5.2 First order

Collecting the first-order terms of Eqs. (12)–(20), dividing by $F_A F_B$ and eliminating z_X and ξ_X through (I)–(II) collapses the six terms into Eqs. (M-59)–(M-60),

$$\alpha_1 = \alpha_0\,\mathbb{G}^{-} + \mathbb{E}^{-}\mathfrak{D}, \qquad \varepsilon_1 = -\varepsilon_0\,\mathbb{G}^{+} + \mathbb{E}^{+}\mathfrak{D} \tag{31}$$

The intermediate steps are

$$z_X^{\mp} \equiv z_X \mp \frac{\Delta q_X}{2}\gamma_X = \gamma_X\gamma_X^{\mp} - \frac{\lambda_X}{\Delta q_X}, \qquad \xi_X \pm \frac{1}{2} = \gamma_X\lambda_X - \frac{\gamma_X^{\mp}}{\Delta q_X}, \tag{32}$$

after which the $M_{HT,A}$ group reduces to $\gamma_A\mathbb{G}^{\pm} - \mathbb{E}^{\pm}/\Delta q_A$ and the $M_{HT,B}$ group to $\gamma_B\mathbb{G}^{\pm} + \mathbb{E}^{\pm}/\Delta q_B$. Adding the FC term, which is $+\widetilde{M}_{FC}\mathbb{G}^{-}$ in absorption but $-\widetilde{M}_{FC}\mathbb{G}^{+}$ in emission, assembles α_0 and $-\varepsilon_0$ respectively. The relative sign between the two lines of Eq. (31) is therefore not a matter of convention: the HT groups retain their orientation in both branches while the FC bracket reverses, which is the FC–HT parity reversal of Eq. (17) carried to first order.

Equation (31) may equivalently be resolved mode by mode,

$$\mathbb{E}^{\pm}\mathfrak{D} = M_{HT,A}\,\mathscr{U}_A^{\pm} - M_{HT,B}\,\mathscr{U}_B^{\pm}, \qquad \mathscr{U}_X^{\pm} = \frac{\gamma_X\gamma_Y^{\pm} - \lambda_A\lambda_B}{\Delta q_X} \pm \frac{\gamma_Y^{\pm}}{2}, \qquad (Y \neq X). \tag{33}$$

Both $\mathbb{G}^{\pm}$ and $\mathbb{E}^{\pm}\mathfrak{D}$ are antisymmetric under $A \leftrightarrow B$, so the slopes change sign when the two coordinates are exchanged, as they must for a rotation whose sense is reversed.

For $u_A = u_B = 0$, Eq. (27) gives $\lambda_X = -\gamma_X^+$ and hence

$$\mathbb{G}^+ = 0, \qquad \mathbb{G}^- = \gamma_A^+\gamma_B^- - \gamma_A^-\gamma_B^+ = \Delta\nu_A \frac{\Delta q_B}{\Delta q_A} - \Delta\nu_B \frac{\Delta q_A}{\Delta q_B}, \tag{34}$$

so that the emission slope loses its ε_0 term entirely.

5.3 The asymmetry to first order

Substituting Eq. (28) into the definition of the asymmetry factor, Eq. (M-10), and expanding to first order in θ gives $\zeta \simeq \zeta^{(0)} + \theta\zeta^{(1)}$ with

$$\zeta^{(1)} = \frac{4\alpha_0\varepsilon_0\left(\varepsilon_0\alpha_1 - \alpha_0\varepsilon_1\right)}{\left(\alpha_0^2 + \varepsilon_0^2\right)^2}. \tag{35}$$

Inserting Eq. (31) and using $\mathbb{G}^- + \mathbb{G}^+ = 2\mathbb{G}$, where $\mathbb{G} = \lambda_B\gamma_A - \lambda_A\gamma_B$ is the unshifted bracket,

$$\varepsilon_0\alpha_1 - \alpha_0\varepsilon_1 = 2\alpha_0\varepsilon_0\,\mathbb{G} + \mathfrak{D}\left(\varepsilon_0\mathbb{E}^- - \alpha_0\mathbb{E}^+\right), \tag{36}$$

which on substitution into Eq. (35) gives Eq. (M-61). The slope therefore carries two contributions: a nuclear one weighted by $\mathbb{G}$, which survives at $\mathfrak{D} = 0$, and an HT-weighted one carried by $\mathfrak{D}$. Both share the prefactor $\alpha_0\varepsilon_0$, which vanishes whenever one member of the cross-swap pair is extinguished, $|\kappa| \to 1$.

6 Regimes of the Duschinsky-induced asymmetry

Within the two-mode subspace each mode is either a participant, $\Delta\nu_X \neq 0$, or a spectator, $\Delta\nu_X = 0$; and independently either displaced, $\Delta q_X \neq 0$, or undisplaced. An all-spectator band is mirror-symmetric to all orders. Two undisplaced participants leave the band forbidden at $\theta = 0$ and at first order, so that Eq. (35) does not apply; neither case is considered further.

6.1 Pure Franck–Condon

When $M_{HT,A} = M_{HT,B} = 0$ the transition is purely FC, so $\kappa = 0$, $\mathfrak{D} = 0$ and $\zeta^{(0)} = 0$; the amplitudes reduce to $\alpha_0 = \varepsilon_0 = \widetilde{M}_{FC}$ with $\alpha_1 = \widetilde{M}_{FC}\mathbb{G}^-$ and $\varepsilon_1 = -\widetilde{M}_{FC}\mathbb{G}^+$. Equation (35) then gives Eq. (M-62),

$$\zeta^{(1)} = \mathbb{G}^- + \mathbb{G}^+ = 2\left(\lambda_B\gamma_A - \lambda_A\gamma_B\right), \tag{37}$$

and for both modes in their vibrational ground states Eq. (34) makes this even cleaner:

$$\zeta^{(1)} = \Delta\nu_A \frac{\Delta q_B}{\Delta q_A} - \Delta\nu_B \frac{\Delta q_A}{\Delta q_B} \tag{38}$$

No electronic prefactor survives. Equation (37) requires both modes to be displaced and at least one to be a participant, since $\gamma_X = 0$ for a spectator while $\lambda_X = 0$ for an undisplaced mode; two displaced spectators give $\zeta^{(1)} = 0$.

6.2 An undisplaced participant

Let mode A be a participant but undisplaced, $\Delta q_A \to 0$. Then $F_A \to 0$ by orthogonality, so the reduction of Eq. (28) by $F_A F_B$ is singular and the amplitudes must instead be normalised by $H_A F_B$. Every quantity remains finite in this normalisation, and in the equal-frequency harmonic limit the band retains intensity only for $|\Delta\nu_A| = 1$ with $M_{HT,A} \neq 0$.

At $\Delta q_A = 0$ both Z_A and Ξ_A vanish, and the only surviving mode-A quantity is the ratio $\rho_A = \Lambda_A/H_A = \lambda_A/\gamma_A$. Identity (III) fixes it since the Δq_A-proportional terms vanish, leaving $\lambda_A = -\gamma_A$ for $\Delta\nu_A > 0$ and $+\gamma_A$ for $\Delta\nu_A < 0$ such that

$$\rho_A = -\frac{1}{\Delta\nu_A}, \qquad |\Delta\nu_A| = 1. \tag{39}$$

Here $\zeta^{(0)} = 0$, so the asymmetry is entirely first order in θ. Carrying the expansion through with the mode-A values above gives

$$\zeta^{(1)} = \frac{2M_{HT,B}}{M_{HT,A}}\left(\Delta\nu_A z_B + \xi_B\right) - \Delta\nu_A \frac{\widetilde{M}_{FC}}{M_{HT,A}}\Delta q_B, \tag{40}$$

in which $\Delta\nu_A = \pm 1$ enters as a sign throughout. Eliminating z_B and ξ_B with (I)–(II) and factorising,

$$\Delta\nu_A z_B + \xi_B = \left(\Delta\nu_A\gamma_B + \lambda_B\right)\left(\gamma_B - \frac{\Delta\nu_A}{\Delta q_B}\right) = -\left(\Delta\nu_A\gamma_B + \lambda_B\right)\frac{\Delta\nu_A + \Delta\nu_B}{\Delta q_B}, \tag{41}$$

using $\gamma_B \Delta q_B = -\Delta\nu_B$, which gives Eq. (M-63). The limit is attained for $|\Delta q_A| \ll |M_{HT,A}/\widetilde{M}_{FC}|$, the displacement at which the FC intensity of mode A becomes comparable to its HT intensity.

Equation (41) carries the selection rule of Eq. (M-64): the HT pathway closes entirely when $\Delta\nu_A + \Delta\nu_B = 0$, that is when the combination band conserves the total number of vibrational quanta. The asymmetry is then $\zeta^{(1)} = -\Delta\nu_A(\widetilde{M}_{FC}/M_{HT,A})\Delta q_B$, independent of $M_{HT,B}$.

For $\Delta\nu_A = +1$ and $\Delta\nu_B \geq 0$, substituting (III) into (41) gives $z_B + \xi_B = (\Delta\nu_B + 1)(\frac{1}{2} + R_B)$, so that

$$\zeta^{(1)} = \frac{2M_{HT,B}}{M_{HT,A}}\big(\Delta\nu_B + 1\big)\Big(\tfrac{1}{2} + R_B\Big) - \frac{\widetilde{M}_{FC}}{M_{HT,A}}\Delta q_B, \tag{42}$$

which for $u_B = 0$, where $R_B = 0$, reduces to

$$\zeta^{(1)} = \frac{M_{HT,B}}{M_{HT,A}}\big(1 + \Delta\nu_B\big) - \frac{\widetilde{M}_{FC}}{M_{HT,A}}\Delta q_B. \tag{43}$$

Equation (42) is restricted to $\Delta\nu_B \geq 0$, since (III) is; the factorised form (41) is unrestricted.

When the partner is an undisplaced spectator, $\Delta\nu_B = \Delta q_B = 0$, the FC term vanishes and $\xi_B = 0$ by consequence (ii), leaving $z_B = u_B + \frac{1}{2}$, and hence

$$\zeta^{(1)} = \Delta\nu_A \frac{M_{HT,B}}{M_{HT,A}}\big(2u_B + 1\big), \tag{44}$$

which is Eq. (M-65).

6.3 A displaced participant

When a participant is displaced it carries FC intensity in its own right and the single-mode FC–HT interference gives $\zeta^{(0)} \neq 0$ already at the AS level. If the partner is also displaced, no special structure survives and one recovers the general result, Eqs. (35)–(36).

With the spectator undisplaced, the exact block values of Eq. (26) may be substituted directly into Eqs. (12)–(20); no limiting process is involved. Every term carrying Λ_B or H_B vanishes, and $\Xi_B = 0$, $F_B = 1$, $Z_B = u_B + \frac{1}{2}$ leave only

$$\alpha_1 = M_{HT,B}\Big[\tfrac{1}{2}\gamma_A^- - \big(u_B + \tfrac{1}{2}\big)\lambda_A\Big], \qquad \varepsilon_1 = -M_{HT,B}\Big[\tfrac{1}{2}\gamma_A^+ + \big(u_B + \tfrac{1}{2}\big)\lambda_A\Big], \tag{45}$$

with $\alpha_0 = \widetilde{M}_{FC} + M_{HT,A}\gamma_A$ and $\varepsilon_0 = \widetilde{M}_{FC} - M_{HT,A}\gamma_A$, as in Eq. (M-66). Substituting into Eq. (35),

$$\zeta^{(1)} = \frac{2M_{HT,B}\,\alpha_0\varepsilon_0}{(\alpha_0^2 + \varepsilon_0^2)^2}\Big\{\alpha_0\big[\gamma_A^+ + (2u_B + 1)\lambda_A\big] + \varepsilon_0\big[\gamma_A^- - (2u_B + 1)\lambda_A\big]\Big\} \tag{46}$$

which is Eq. (M-67). The same expression follows from the general result, Eq. (M-61), in the limit $\Delta q_B \to 0$: there $\mathbb{G} \to 0$ while $\mathbb{E}^\pm \to 0$ and $\mathfrak{D} \to \infty$ with $\mathbb{E}^\pm\mathfrak{D}$ finite, the two agreeing with a residual of order Δq_B. The direct substitution is preferable because it is exact at $\Delta q_B = 0$, where $\lambda_B/\Delta q_B$ and $\mathfrak{D}$ are individually undefined.

For $u_A = 0$, Eq. (27) gives $\lambda_A = -\gamma_A^+$ and the bracketed factors become explicit in $\Delta\nu_A$ and Δq_A. Equation (46) is strictly proportional to $M_{HT,B}$, so $\zeta^{(1)} = 0$ when the undisplaced spectator is HT-inactive; and the prefactor $\alpha_0\varepsilon_0$ suppresses the correction as $\zeta^{(0)}$ saturates towards ± 1.
